# Advances in Kiwifruit Postharvest Management: Convergence of Physiological Insights, Omics, and Nondestructive Technologies

**DOI:** 10.3390/cimb48010009

**Published:** 2025-12-22

**Authors:** Shimeles Tilahun, Min Woo Baek, Jung Min Baek, Han Ryul Choi, DoSu Park, Cheon Soon Jeong

**Affiliations:** 1 Agriculture and Life Science Research Institute, Kangwon National University, Chuncheon 24341, Republic of Korea; 2 Department of Horticulture, Kangwon National University, Chuncheon 24341, Republic of Korea; 3 Department of Horticulture and Plant Sciences, Jimma University, Jimma 378, Ethiopia; 4 Interdisciplinary Program in Smart Agriculture, Kangwon National University, Chuncheon 24341, Republic of Korea; 5 Research and Development Center, Life & Technology Co., Ltd., Pyeongtaek-si 17700, Republic of Korea

**Keywords:** transcriptomics, metabolomics, biomarkers, postharvest quality, nondestructive technologies

## Abstract

Kiwifruit (*Actinidia* spp.) is valued for its sensory quality and nutritional richness but faces postharvest challenges such as rapid softening, chilling injury, and physiological disorders. Conventional management strategies help maintain quality yet insufficient to capture the complexity of ripening, stress physiology, and cultivar-specific variation. Recent research emphasizes the continuum from preharvest to postharvest, where orchard practices, harvest maturity, and handling conditions influence quality and storage potential. Omics-driven studies, particularly transcriptomics and metabolomics, have revealed molecular networks regulating softening, sugar–acid balance, pigmentation, antioxidant properties, and chilling tolerance. Integrated multi-omics approaches identify key biomarkers and gene–metabolite relationships linked to ripening and stress responses. Complementing omics, nondestructive estimation technologies, including hyperspectral imaging, near-infrared spectroscopy, acoustic profiling, and chemometric models are emerging as practical tools for real-time classification of maturity, quality, and storability. When calibrated with omics-derived biomarkers, these technologies provide predictive, non-invasive assessments that can be deployed across the supply chain. Together, the convergence of postharvest physiology, omics, and nondestructive sensing offers a pathway toward precision quality management and sustainable kiwifruit production. This review synthesizes recent advances across these domains, highlighting mechanistic insights, practical applications, and future directions for integrating omics-informed strategies with commercial postharvest technologies.

## 1. Introduction

Kiwifruit (*Actinidia* spp.) has emerged as one of the most economically and nutritionally significant horticultural crops, widely cultivated across Asia, Europe, New Zealand (Oceania), and the Americas [1]. Its commercial success stems not only from unique sensory properties but also from its high concentrations of vitamin C, phenolics, flavonoids, carotenoids, and other health-promoting metabolites that make it a functional food [1,2]. However, maintaining fruit quality during long storage and global distribution presents formidable postharvest challenges, including chilling injury, softening, and pathogen susceptibility [3,4]. These physiological constraints have driven extensive research into optimizing harvest maturity, storage conditions, and handling practices [5,6,7,8].

Recent advances in postharvest research emphasize the continuum from preharvest to postharvest management, where field-level practices such as agro-ecological choice, chitosan or melatonin spraying, and harvest timing directly influence storability and consumer quality [4,7,9]. In addition, controlled atmosphere storage, temperature management, biopreservation strategies, particularly the application of beneficial microorganisms (e.g., antagonistic yeasts and bacteria) and natural elicitors that stimulate host defense responses, and chemical treatments such as 1-methylcyclopropene (1-MCP) and jasmonates (JAs) have been extensively investigated to mitigate postharvest softening, chilling sensitivity, and disease incidence [10,11,12]. Despite such progress, the complex interplay of ripening, stress physiology, cultivar-specific traits, harvest maturity, and storage conditions cannot be fully addressed by conventional physicochemical indices alone, highlighting the need for omics-driven and nondestructive approaches to provide deeper mechanistic insights into postharvest management.

In this context, omics technologies (transcriptomics, metabolomics, proteomics, and their integration) provide transformative insights into the molecular and metabolic basis of fruit development, ripening, and stress responses [1,13,14]. Beyond broad conceptual advances, specific transcriptomic studies have shed light on gene regulatory networks that govern postharvest behavior. For instance, investigations have identified genes and pathways linked to softening, sugar–acid metabolism, ethylene biosynthesis and signaling, antioxidant defense, and cultivar-dependent responses [15,16,17,18]. In parallel, metabolomic approaches complement these insights by profiling dynamic shifts in primary and secondary metabolites across cultivars, tissues, and developmental stages [19,20,21,22,23]. These metabolomic approaches also clarify dynamic responses during ripening and storage [2,16,24] and reveal how treatments such as bagging, chitosan, methyl jasmonate (MeJA), 1-MCP, ozone, blue light, and coatings reprogram sugar metabolism, cell wall disassembly, lipid turnover, and antioxidant defenses [17,25,26,27,28,29,30]. In addition, metabolomics has identified stress-tolerance markers, linking flavonoid metabolism, lipid peroxidation, and energy balance to differences in chilling or anaerobic tolerance [15,31,32,33], and has guided breeding by distinguishing germplasm and ploidy variants with superior profiles of anthocyanins, carotenoids, or vitamin C [20,34,35].

More recently, multi-omics integration has emerged as a transformative approach in kiwifruit research for detailed gene–metabolite networks, linking transcriptional regulators with metabolic pathways for sugars, flavonoids, anthocyanins, proanthocyanidins, and vitamin C, thereby clarifying how key transcription factors coordinate nutritional and sensory quality traits [16,21,35,36]. Multi-omics has also revealed how storage and treatment interventions such as calcium, chitosan coatings, blue light, ethanol fumigation, and riboflavin photosensitization modulate lipid metabolism, cell wall disassembly, energy pathways, and antioxidant defenses, offering mechanistic explanations for delayed ripening and enhanced disease resistance [27,29,37,38,39]. Beyond ripening and treatments, multi-omics has been applied to abiotic stress responses, such as freezing, chilling, and cold-chain fluctuations, where it identified metabolic markers like flavonoids, lipids, and organic acids, and regulatory hubs like HsfA3a that govern resilience or susceptibility across genotypes [15,31,40]. Importantly, integration across omics has provided novel biomarkers for harvest maturity and storability, exemplified by candidate transcripts and metabolites predictive of storage disorder [41], offering practical potential for supply-chain management. Moreover, resources like the Kiwifruit PanGenome Database (KPGD) have expanded opportunities for trait characterization, enabling the integration of structural variants, SNPs, and transcriptomic datasets into multi-omics pipelines for comparative genomics and molecular breeding [42]. Although many studies remain correlative and cultivar-specific, the accumulating evidence highlights that multi-omics not only deepens mechanistic insight but also provides tangible tools for developing precision breeding, optimized storage, and eco-friendly postharvest strategies in kiwifruit.

Complementing these biological insights, nondestructive quality estimation technologies are transforming how fruit quality is monitored across the supply chain. Portable tools such as near-infrared (NIR) sensors, smartphone-based platforms, and LED–photodiode devices now enable rapid, low-cost, and real-time assessment of fruit maturity and internal quality, reducing reliance on destructive sampling [43,44]. Advances in imaging, spectroscopy, and machine learning have also significantly improved accuracy in detecting defects, predicting ripeness, and classifying textural properties under commercial conditions [45,46,47]. Recent studies demonstrate that combining multiple sensing modalities such as hyperspectral imaging (HIS) with Fourier transform near-infrared (FT-NIR) spectroscopy, fluorescence imaging with machine learning, or volatile analysis with HSI further enhances robustness and predictive power [48,49,50]. Collectively, these technologies are moving beyond research settings toward practical application, offering scalable, non-invasive solutions for growers, distributors, and retailers. When integrated with omics-derived biomarkers and mechanistic models, nondestructive sensing holds strong potential to support precision harvest decisions, optimize storage and transport, and deliver consistent fruit quality to consumers.

Taken together, the convergence of postharvest physiology and technology, omics, and nondestructive estimation provides an unprecedented opportunity to advance sustainable kiwifruit production, storage, and distribution. Nevertheless, key challenges remain in translating laboratory findings into commercial practices, integrating multi-omics datasets with sensor platforms, and validating biomarkers across cultivars, environments, and seasons. This review therefore synthesizes recent progress in kiwifruit postharvest physiology and technology, transcriptomics, metabolomics, integrated omics, and nondestructive quality estimation, with the goal of outlining mechanistic insights and practical strategies that can inform both breeding programs and industry adoption. To ensure transparency and minimize selection bias, the review followed the preferred reporting items for systematic reviews and meta-analyses (PRISMA) guidelines. A structured literature search was conducted in Web of Science, Scopus, PubMed, and ScienceDirect for studies published between 2016 and 2025. The search initially retrieved 180 records, of which 30 duplicates were removed. The remaining 150 articles were screened by title and abstract, and 130 were subjected to full-text evaluation. Ultimately, 126 peer-reviewed studies met the inclusion criteria and were incorporated into this review. Eight earlier foundational works (2016–2019) were retained to provide historical and mechanistic context, while the majority of included studies (118) were published from 2020 onward, reflecting the rapid expansion of omics-driven research and nondestructive sensing technologies in kiwifruit. This temporal distribution confirms that the evidence synthesized here is strongly anchored in the most recent decade of postharvest and molecular innovation.

## 2. Postharvest Physiology and Technology

Kiwifruit is highly perishable, and its storability is constrained by complex physiological and technological challenges such as rapid softening, chilling injury, lignification, pathogen-induced decay, and uneven ripening. To address these, research has focused on understanding key physiological processes, optimizing harvest timing, and developing storage and treatment technologies ranging from controlled atmosphere and temperature management to chemical, biological, and eco-friendly alternatives.

### 2.1. Key Postharvest Challenges

Kiwifruit is highly perishable, with its postharvest life constrained by multiple challenges including rapid softening [3,10], chilling injury [6,31], lignification [51], pathogen-induced soft rot [12,52], off-flavor development such as ethanol fermentation [53], and uneven ripening [5,54]. These physiological and technological constraints ultimately threaten storability and consumer acceptability. Xia et al. [3] summarized postharvest strategies into physical, chemical, and biological categories, each with inherent limitations. Physical methods, including low temperature, controlled atmosphere (CA), ozone, and heat treatments, can delay senescence but may induce chilling injury or impose high-energy costs. Chemical treatments such as 1-MCP, nitric oxide (NO), melatonin, MeJA, and essential oils are effective at suppressing ethylene action and oxidative damage but raise concerns regarding concentration and consumer safety. Biological approaches, including the use of antagonistic microbes, offer eco-friendly disease control but remain inconsistent across cultivars and environments.

Recent mechanistic studies have further revealed that mechanical stress itself represents a hidden yet significant postharvest challenge. Compression [55] and puncture [56] injuries accelerate ethylene production, respiration, and cell wall degradation, thereby triggering stress-induced softening. Such findings highlight that ‘damage physiology’ can be as critical to postharvest quality as chilling or ethylene physiology. Lignification during cold storage remains another major constraint to kiwifruit quality, as it reduces palatability and consumer acceptance. Li et al. [51] reported that while 1-MCP delayed softening, it aggravated lignin deposition in ‘Xuxiang’, whereas MeJA and methyl salicylate mitigated lignification by suppressing phenylpropanoid-related enzymes. Chilling-induced lignification has likewise been associated with the activation of phenylpropanoid metabolism, linking stress responses to textural decline. In parallel, disturbances in energy metabolism represent an additional postharvest challenge, as ATP depletion and membrane instability accelerate senescence and quality loss. Huang et al. [10] demonstrated that 1-MCP treatment could mitigate this problem by preserving ATP pools, stabilizing membrane fatty acid composition, and maintaining membrane integrity, thereby delaying senescence. Collectively, these findings illustrate that postharvest challenges arise from interconnected physiological disorders, including metabolic imbalances, structural deterioration such as cell wall modification and lignin deposition, and weakened defense systems against oxidative stress and pathogen attack.

### 2.2. Preharvest to Postharvest Continuum

Agro-ecological conditions, preharvest management practices, and harvest maturity strongly influence kiwifruit quality. Ozturk et al. [7] demonstrated striking regional differences in ‘Hayward’ across Türkiye, where Yalova-grown fruits had shown better storability, highest vitamin C, and best antioxidant activity, whereas firmness retention and flavonoid profiles varied in Giresun and Ordu. These environment–cultivar interactions highlight that growing conditions play a decisive role in determining both the storability and the nutritional attributes of kiwifruit. Harvest timing is equally critical, as Choi et al. [5] showed that earlier harvest (160 DAFB) maintained firmness and reduced decay over four months, whereas later harvests promoted higher respiration, ethylene, and softening. However, optimal eating quality diverged by cultivar, with gold ‘Haegeum’ and red ‘Hongyang’ types reaching consumer-preferred quality earlier than green ‘Hayward’. Burdon et al. [8] re-examined the traditional 6.2% soluble solids content (SSC), harvest index, showing that early increases in SSC may reflect soluble sugar translocation into the fruit rather than starch degradation. They concluded that SSC alone does not adequately indicate maturity and must be interpreted within the context of starch metabolism phases and seasonal conditions. Wang et al. [6] refined harvest maturity recommendations for red ‘Hongyang’, showing that fruits at SSC 6.5–7.5% (stage II) possessed higher antioxidant and energy metabolism-related enzyme activities, improving chilling tolerance and ROS homeostasis. Stage I fruits were chilling-sensitive, while stage III fruits decayed rapidly despite chilling resistance. These studies converge on the idea that cultivar-specific SSC or DAFB windows optimize both chilling tolerance and flavor.

The preharvest to postharvest continuum is further highlighted by biostimulant applications. Kusarigama et al. [4] showed that chitosan sprays in ‘Garmrok’ reduced ethylene and respiration, suppressed ripening-related genes, improved antioxidant capacity, and ultimately prolonged storability. Complementing this, Peng et al. [9] reported that low-dose preharvest melatonin enhanced disease resistance and nutritional quality in ‘Guichang’, while X. Yang et al. [40] demonstrated that preharvest MeJA sprays strengthened antioxidant and defense systems more effectively than postharvest treatments, thereby reducing soft rot and preserving aroma. In addition, Lembo et al. [57] demonstrated that orchard sprays of cis-3-hexenyl butyrate enhanced anthocyanin accumulation in red kiwifruit, though at the expense of storage life. At the storage intake stage, Zhang et al. [58] compared four pre-cooling strategies for ‘Hongyang’ and identified forced-air cooling as the most effective, as it rapidly lowered core temperature, preserved firmness, reduced weight loss, and alleviated oxidative stress compared to gradual or delayed cooling. Collectively, these studies underscore that orchard-level interventions and early postharvest handling steps jointly play a decisive role in shaping postharvest storability.

### 2.3. Conventional and Innovative Postharvest Approaches

Conventional cold and CA storage remain the backbone of kiwifruit preservation, yet recent innovations have refined these approaches and introduced practical alternatives. Chai et al. [59] demonstrated that high O_2_/N_2_ CA accelerated ripening while preserving eating quality, offering a non-chemical means of delivering ready-to-eat fruit. Complementing this, Chai et al. [54] showed that low-temperature storage (10 °C) induced starchy off-flavors in ‘Cuixiang’, but transferring fruit to room temperature at the appropriate firmness stage eliminated this defect, providing practical guidelines for temperature management.

Chemical and hormonal treatments remain central to postharvest innovation, though their effects vary across compounds and contexts. Ma et al. [11] reported that combining 1-MCP with postharvest ripening maintained quality more effectively than either treatment alone by stabilizing sugars, acids, antioxidants, and firmness. While numerous studies confirm the efficacy of 1-MCP in delaying senescence and preserving fruit quality, important limitations have been noted: Li et al. [51] showed it aggravates lignification, and Zhao et al. [60] highlighted cultivar- and dose-dependent metabolic responses. Beyond 1-MCP, Yan et al. [61] introduced tenuazonic acid as a novel compound that suppressed cell wall degradation and oxidative stress, thereby maintaining firmness. In contrast, Yan et al. [62] showed that H_2_O_2_ accelerates ripening and flavor development but reduced storability. Additional chemical elicitors have also shown promise, including nitric oxide, which enhanced disease resistance through the stimulation of PAL, POD, and β-1,3-glucanase [63], and riboflavin (photosensitized with blue light), which primed defense against *Botrytis cinerea* [39]. While chemical and hormonal treatments remain dominant, biological approaches are emerging as sustainable alternatives. Hao et al. [64] reported that *Lactiplantibacillus pentosus* CW5 cell-free supernatant delayed softening and reduced decay while shaping the fruit microbiome to suppress pathogens. These findings illustrate how microbial derivatives can improve fruit quality while reducing reliance on synthetic chemicals. Such eco-friendly methods complement traditional fungicide alternatives and align with the broader movement toward residue-free postharvest technologies.

Advances in packaging technologies are also advancing postharvest management. Ding et al. [29] developed visible light-responsive chitosan composite films that suppressed lipid-degrading enzymes and preserved membrane integrity, outperforming conventional polyethylene packaging. At the same time, managing physical stress remains critical, as Polychroniadou et al. [55] and Chen et al. [56] showed that compression and puncture injuries accelerate ripening and quality loss, emphasizing the importance of optimized packaging and transportation systems. While these physiological and technological approaches highlight the visible outcomes of postharvest interventions (Table 1), recent omics-driven studies provide mechanistic depth by uncovering the molecular pathways through which these treatments act.

## 3. Progress in Transcriptomics of Kiwifruit

Transcriptomic research in kiwifruit has undergone a major transformation in the last decade, moving from candidate-gene studies to genome-wide approaches supported by multiple high-quality reference genomes and a pan-genome [42]. Public resources such as the Kiwifruit Genome Database have enabled comparative expression profiling, regulatory network construction, and cross-species gene mining, which in turn have accelerated discovery of pathways controlling fruit ripening, storability, stress tolerance, and organoleptic traits. Recent investigations provide valuable insights into how transcriptomes shift across cultivars, ripening stages, postharvest treatments, and storage conditions, while also identifying key functional genes that regulate physicochemical and antioxidant properties.

### 3.1. Transcriptome Responses to Species and Cultivar

Transcriptomic studies consistently highlight species- and cultivar-specific responses in kiwifruit, with differences in ripening behavior, storability, and nutritional quality often driven by distinct gene regulatory programs. Mitalo et al. [70] compared the yellow-fleshed ‘Sanuki Gold’ (*A. chinensis* var. chinensis) and green-fleshed ‘Hayward’ (*A. deliciosa*), showing that although both cultivars softened, accumulated sugars, and lost acidity in an ethylene-independent manner, their temperature sensitivities differed. ‘Sanuki Gold’ showed rapid softening and strong induction of ripening-associated genes even at 15 °C, whereas ‘Hayward’ required temperature below 10 °C to trigger comparable expression of *AcACO3*, *AcXET2*, *AcPG*, *AcEXP1*, and several transcription factors. Choi et al. [71] reinforced this cultivar difference by comparing ethylene-treated ‘Hayward’ and ‘Haegeum’, finding overlapping induction of cell wall-modifying genes (PGs, pectate lyases, expansins) but the scale and timing of expression differed strongly between cultivars, explaining variation in softening kinetics. Together, these studies show that cultivar behavior reflects both temperature- and ethylene-dependent transcriptional programs, explaining contrasting storability and quality profiles.

Pigmentation pathways further illustrate cultivar-specific variation. Xia et al. [72] reported that the brighter yellow coloration of ‘Jinshi 1’ compared with ‘Hort16A’ was associated with stronger expression of chlorophyll catabolic genes (*CBR*, *PPH*, *PAO*) and the specific accumulation of β-cryptoxanthin, whereas CCD1 and CCD4 were more active in ‘Hort16A’. Similarly, Liang et al. [22] showed that red-fleshed ‘Donghong’ displays stage-specific anthocyanin accumulation regulated by structural genes (*AcLDOX2*, *Ac5GGT1*, *Ac5AT2*) and transcription factors, with *AcbHLH74-2* promoting and *AcMYB4-1* repressing anthocyanin biosynthesis.

Recent studies have expanded the cultivar-specific perspective beyond single traits to encompass broader transcriptomic regulation. Liu et al. [17] compared transcriptomic responses during storage and showed that variation in starch metabolism and phenolic biosynthesis pathways underpin contrasting differences in firmness and antioxidant accumulation. Sun et al. [15] likewise highlighted genotype-dependent expression of ethylene biosynthesis and signaling genes, as well as antioxidant-related pathways, whereas Shu et al. [16] integrated multiple transcriptomic datasets to link cultivar differences in -metabolism with consumer-preferred flavor attributes and variable postharvest performance.

Taken together, these studies demonstrate that cultivar type is a dominant factor shaping transcriptomic landscapes in kiwifruit. Differences in temperature sensitivity, ethylene responsiveness, pigment accumulation, and regulation of cell wall, sugar, and antioxidant metabolism all contribute to the diverse ripening behaviors and storability observed across *Actinidia* species and cultivars. By identifying both common and exclusive regulatory modules, transcriptomic research provides a mechanistic basis for breeding strategies aimed at optimizing storability, sensory quality, and nutritional value in cultivar-specific contexts.

### 3.2. Transcriptome Responses to Ripening Stages

Ripening in kiwifruit is a highly coordinated process involving reprogramming of secondary metabolism, cell wall modification, and hormonal regulation. In the red-fleshed cultivar ‘Donghong’, Liang et al. [22] demonstrated that anthocyanin accumulation peaked at mid-ripening, coinciding with the upregulation of *AcLDOX2* and other phenylpropanoid biosynthetic genes, underscoring the tight regulation of secondary metabolism. Jiayi Zhang et al. [73] expanded this view in ‘Xuxiang’, where ethylene induced about 2000 differentially expressed genes (DEGs), with firmness emerging as the dominant trait shaping transcriptomic changes. Strong induction of pectinesterases, cellulases, and expansins highlighted cell wall breakdown as a central process, while canonical correlation analysis linked sugar and organic acid metabolism to visible ripening traits. Lin et al. [74] further proposed a dual regulatory framework that integrating metabolic and hormonal cues: (i) the T6P-SnRK1-TOR pathway moderating starch metabolism and post-harvest energy status, and (ii) an ABA-CK-ethylene network regulating cell wall degradation and softening. Complementing evidence from Sun et al. [15] and Shu et al. [16] confirmed that transcriptomic reprogramming during ripening consistently involves ethylene signaling, sugar–acid metabolism, and antioxidant pathways. Liu et al. [17] emphasized cultivar-dependent regulation of starch degradation and phenolic metabolism under storage conditions, while Ye et al. [18] identified transcription factor families such as MYB, NAC, and ERF as central hubs linking hormonal crosstalk with ripening traits. Collectively, these studies illustrate that overlapping transcriptional modules integrate sugar remobilization, hormone signaling, and secondary metabolism to govern kiwifruit ripening with cultivar- and stage-specific patterns dictating the ripening progression and fruit quality.

### 3.3. Transcriptome Responses to Postharvest Treatments

Postharvest interventions strongly influence transcriptional networks in kiwifruit, primarily through modulation of hormone signaling and stress-response pathways. Gan et al. [75] demonstrated that ethylene promotes ripening not only through cell wall enzymes but also by accelerating auxin catabolism. Ethylene-induced ethylene response factors (*ERFs*) such as *AcERF1B* and *AcERF073* directly activated *AcGH3.1*, which conjugates free IAA into IAA-Asp, thereby reducing free auxin and promoting softening. Choi et al. [71] and Jiayi Zhang et al. [73] reinforced the central role of ERFs, showing that they regulate sugar metabolism, respiration, and firmness-related processes, confirming their position as transcriptional hubs in ethylene-driven ripening. In contrast, antagonistic treatments often suppress these ethylene-centered pathways. Yang et al. [76] found that nitric oxide (NO) fumigation downregulated genes encoding pectin-degrading enzymes (PG, PL, PE) and β-galactosidases while repressing ethylene biosynthesis genes, simultaneously upregulating cellulose synthases to maintain wall integrity. Similarly, Lin et al. [74] revealed that abscisic acid (ABA) and cytokinin (CK) interact synergistically with ethylene to accelerate cell wall breakdown, suggesting that postharvest treatments operate within overlapping networks that can be either enhanced or repressed.

Other hormonal and stress-related interventions further diversify transcriptomic outcomes. Niu et al. [31] showed that MeJA alleviates chilling injury by suppressing ethylene biosynthetic genes (*ACS*, *ACO*) and cell wall-modifying genes (*PG*, *PL*, *PE*), while sustaining antioxidant defenses and downregulating stress-related transcription factors (*MYB*, *WRKY*, *bHLH*, *bZIP*). Xu et al. [27] demonstrated that blue light delays ripening of ‘Jinyan’ by downregulating ethylene synthesis genes (*ACS1*, *ACO1/2/5*), starch degradation genes (*AMY1*, *BAMs*), and cell wall-modifying genes (*PG*, *PME*, *PL*, *XTH*, *β-Gal*), with parallel shifts in light-responsive *MYB* and *bHLH* transcription factors. Luo et al. [66] highlighted that melatonin (MT) maintains vitamin C content by upregulating biosynthesis and recycling genes (*AcGME2*, *AcGalDH*, *AcGalLDH*, *AcGR*, *AcDHAR*, *AcMDHAR1*) while repressing degradation genes (*AcAO*), linking MT to both antioxidant stability and delayed senescence. Likewise, Yan et al. [67] reported that exogenous γ-aminobutyric acid (GABA) treatment suppressed the expression of starch-degrading (*BAMs*, *PHS*) and cell wall-modifying genes (*PGs*, *expansins*, *XTHs*), thereby preserving firmness during long-term cold storage. Moreover, multi-hormonal strategies are emerging as a frontier in postharvest regulation. He et al. [68] tested a plant endogenous hormone complex (brassinolide, melatonin, MeJA, and salicylic acid); showing that combined treatments more effectively preserved firmness, vitamin C, and organic acids, while downregulating ethylene biosynthesis genes (*ACO* family) compared to single-hormone applications. Similarly, Qi et al. [69] demonstrated that forchlorfenuron (CPPU) treatments induced dose- and temperature-dependent transcriptomic changes in sugar, acid, tannin, and antioxidant metabolism, revealing both quality-enhancing and senescence-promoting effects.

Overall, these studies highlight that postharvest treatments act by rewiring transcriptional networks, often centering on ethylene–auxin–ABA crosstalk, but extending to jasmonate, light, melatonin, GABA, and synthetic regulators. This complexity explains why treatments can have divergent outcomes across cultivars and storage conditions, while also providing mechanistic targets for designing integrated, cultivar-specific postharvest strategies.

### 3.4. Transcriptome Responses to Storage

Storage imposes unique transcriptional reprogramming, often linked to stress and quality deterioration. Huan et al. [77] compared ‘Bruno’ and ‘White’ during ambient storage and showed that the alcoholic off-flavor in ‘Bruno’ results from coordinated upregulation of starch-degrading enzymes (β-amylases, invertases), sucrose metabolism genes (sucrose synthases), and fermentation genes (pyruvate decarboxylase, alcohol dehydrogenase). By contrast, ‘White’ maintained lower respiration and avoided ethanol build-up, illustrating cultivar-dependent transcriptional sensitivity to storage stress. Temperature further shapes transcriptional outcomes. Gambi et al. [78] analyzed de-greening in ‘Zesy003’, ‘Zesh004’, and ‘Hayward’, demonstrating that high-temperature storage strongly upregulated chlorophyll catabolic genes (*SGR2*, *PAO1*) in yellow-fleshed cultivars, while ‘Hayward’ showed minimal response. This reveals that storage temperature modulates chlorophyll breakdown in a cultivar-dependent manner.

Transcriptomic studies on stress tolerance have highlighted sugar metabolism as a central factor governing storability. Sun et al. [15] showed that freezing-tolerant *Actinidia arguta* (KL) activated genes linked to trehalose synthesis (*TPS5*), starch degradation (*BAM3.1*), and cold-response transcription factors (*CBFs*, *MYCs*, *MYBs*), whereas the sensitive *A. arguta* cv. Ruby-3 (RB) failed to activate these pathways. Similarly, Gong et al. [79] identified *AcBAM5* and *AcBAM13* as key β-amylase genes in starch degradation during storage, while Qiang et al. [80] mapped 102 invertase genes, many correlated with sugar accumulation across cultivars, highlighting *AaINV* genes as additional sugar-modulating hubs. Complementary transcriptomic evidence links storage interventions to quality preservation. Xu et al. [27] found that blue light treatment suppressed ethylene synthesis and downregulated starch/cell wall genes during ‘Jinyan’ storage, delaying softening. Similarly, Niu et al. [31] demonstrated that methyl jasmonate mitigated chilling injury by repressing ripening-related genes and stress-responsive transcription factors while sustaining antioxidant pathways. Ying et al. [33] further showed that short-term anaerobic treatment enhanced antioxidant gene activity, delaying softening and decay in kiwiberry under cold storage. Collectively, these findings highlight sugar metabolism, particularly the roles of β-amylases and invertases, along with ethylene and antioxidant regulation, as central transcriptional determinants shaping storage performance.

### 3.5. Functional Genes Regulating Physicochemical Properties

Beyond broad transcriptome shifts, several studies have identified specific genes that directly influence physicochemical traits, including texture, sweetness, and mineral composition. Shan et al. [81] reported that *AcKUP2*, a potassium transporter gene in kiwifruit, is localized to the plasma membrane, induced by ethylene, and directly regulated by *AcERF15*, thereby contributing to potassium accumulation and ethylene-mediated postharvest ripening. Complementing this, carbohydrate metabolism has emerged as a central determinant of physicochemical quality. Gong et al. [79] identified *AcBAM5* and *AcBAM13* as key β-amylase genes driving starch breakdown during storage, validated through virus-induced gene silencing experiments that confirmed their causal role in sugar release. Similarly, Qiang et al. [80] mapped 102 invertase genes, of which several genes including *AcINV2, AcINV17, AcINV43* were strongly correlated with sucrose, glucose, and fructose accumulation, linking invertase activity with soluble solids content and consumer-perceived sweetness. In parallel, other transcriptomic investigations reinforce the importance of structural remodeling (cell wall and sugar metabolism) genes in defining firmness and taste. Choi et al. [71] showed that ethylene induced the expression of cell wall-modifying genes, including *AcPG*, *AcPL*, and *AcEXP1*, with cultivar-dependent variation between ‘Hayward’ and ‘Haegeum’. Similarly, Jiayi Zhang et al. [73] found that transcriptomic changes associated with firmness in ‘Xuxiang’ were marked by strong induction of PE and CEL genes, underscoring cell wall disassembly as a genetically controlled process. Together, these findings show that gene networks controlling ion transport, starch and sugar metabolism, and cell wall modification actively govern physicochemical quality in kiwifruit, positioning these functional genes as prime targets for breeding and postharvest strategies to enhance firmness, sweetness, and storability.

### 3.6. Functional Genes Regulating Antioxidant Properties

Secondary metabolism contributes decisively to kiwifruit’s sensory quality and nutritional value, and transcriptomic studies have identified both structural genes and regulatory factors involved in these processes. Zhang et al. [82] combined GC-MS with co-expression analysis and identified three ester biosynthetic genes, *AdFAD1*, *AdALDH2*, and *AdAT17* that strongly correlated with aroma formation. Their expression was regulated by two transcription factors: *AdNAC5* (an activator) and *AdDof4* (a repressor), highlighting the transcriptional control of volatile biosynthesis. Liang et al. [22] also characterized pigmentation and phenolic pathways in red-fleshed ‘Donghong’, identifying epicatechin as the dominant phenolic and linking anthocyanin accumulation to key biosynthetic genes (*AcLDOX2*, *Ac5GGT1*, *Ac5AT2*). They further revealed that transcriptional regulation involved *AcbHLH74-2* as an activator and *AcMYB4-1* as a repressor, underscoring the activator-repressor dynamics that govern anthocyanin accumulation. In addition, Xia et al. [72] showed that the deeper yellow color of ‘Jinshi 1’ compared to ‘Hort16A’ reflected higher expression of *CHYB1* and reduced activity of degradation genes such as *CCD1* and *CCD4*. These shifts in gene expression explain the cultivar-specific accumulation of β-cryptoxanthin, which contributes to both color intensity and provitamin A content. More broadly, studies of secondary metabolism across cultivars and ripening stages [19,20,21] consistently highlight flavonoid- and carotenoid-related transcripts as key regulators of antioxidant properties. Yu et al. [19] identified 125 metabolites across red- and purple-fleshed kiwifruit/kiwiberry cultivars, linking the enhanced flavonol and anthocyanin accumulation in red-fleshed ‘Hongyang’ and purple-fleshed ‘Mini Amethyst’ to structural genes (*AcF3H*, *AcF3′H*, *AcDFR*, *AcF3GT*, *AaF3H*, *AaF3GT*) and transcription factors (*AcMYB10*, *bHLH5*, *AaMYB110*). Mao et al. [35] showed that carotenoid differences between yellow- and green-fleshed cultivars were regulated by *PSY*, *LCYB*, and *CHYB1*, with higher *CHYB1* expression linked to elevated β-cryptoxanthin in yellow-fleshed fruit. Similarly, Jia et al. [20] profiled metabolite–gene associations across multiple *Actinidia* species and identified co-expression of *DFR*, *ANS*, and *UFGT* with anthocyanin accumulation in red-fleshed cultivars, establishing clear molecular bases for variation in color and antioxidant capacity.

Hormonal crosstalk also intersects with antioxidant regulation. For example, MeJA treatments have been shown to activate phenylpropanoid genes (*PAL*, *C4H*, *4CL*) and transcription factors like *MYC2* while repressing *JAZ* repressors, thereby enhancing phenolic and flavonoid accumulation [12,40]. Similarly, melatonin application upregulated *AcGME2*, *AcGalDH*, and *AcMDHAR1*, while downregulating degradation genes such as *AcAO*, maintaining ascorbic acid pools and redox homeostasis during storage [66]. These findings demonstrate that regulatory genes controlling antioxidant metabolism are closely integrated with hormone signaling pathways that modulate stress tolerance. Together, these studies underscore that antioxidants and flavor-related traits in kiwifruit are coordinated by transcription factors acting on structural biosynthetic genes for volatiles, anthocyanins, carotenoids, phenolics, and ascorbic acid. This integrated regulation explains cultivar- and treatment-specific differences in nutritional and sensory qualities, while pinpointing candidate genes and regulatory hubs for breeding and postharvest interventions. Notably, the convergence of hormone signaling with secondary metabolism suggests that transcriptional regulation of antioxidants contributes not only to sensory quality but also to defense against chilling, oxidative stress, and pathogen infection, directly linking antioxidant gene networks to postharvest storability. Table 2 summarizes the transcriptomic studies in kiwifruit that associate cultivar difference, ripening stages, postharvest treatments, and storage responses with functional genes regulating physicochemical properties and antioxidant capacity.

## 4. Advances in Metabolomics of Kiwifruit

Metabolomic studies have greatly enriched our understanding of kiwifruit biology, offering detailed insights into how genotype, developmental stage, storage conditions, and postharvest treatments shape fruit biochemistry. These approaches have identified a wide array of metabolites, including sugars, organic acids, amino acids, volatiles, phenolics, carotenoids, and vitamins, that collectively underpin sweetness, acidity, aroma, coloration, antioxidant activity, and storability. By mapping these metabolic shifts across cultivars, maturity and ripening stages, and stress conditions, metabolomics provides a powerful lens for dissecting the biochemical basis of fruit quality. This section reviews advances in four key areas: (i) species- and cultivar-specific metabolic profiles, (ii) changes during fruit development and ripening, (iii) responses to storage conditions, and (iv) metabolomic shifts following postharvest treatments.

### 4.1. Metabolite Profiling Across Species and Cultivars

Comparative metabolomics has consistently shown that kiwifruit species and cultivars differ markedly in their nutritional and bioactive composition, highlighting the genetic basis of quality variation. Leontowicz et al. [83] demonstrated that hardy kiwifruit *A. arguta*, cv. ‘M1’ contains substantially higher levels of vitamin C, polyphenols, flavonoids, carotenoids, tannins, and dietary fiber than *A. deliciosa* ‘Hayward’, while *A. eriantha* ‘Bidan’ exceeded both in antioxidant activity. Extending this, Huang et al. [84] reported that *A. eriantha* ‘Ganmi No. 6’ accumulated exceptionally high concentrations of gallic acid, reinforcing *A. eriantha* as a metabolically superior germplasm. Similarly, Li et al. [85] compared *A. arguta* ‘Kuilv’, *A. chinensis* ‘Hongyang’, and *A. deliciosa* ‘Hayward’, showing that ‘Kuilv’ combined short development with high vitamin C and sucrose but relatively low glucose and fructose; ‘Hayward’ produced large fruits with abundant glucose and fructose but lower vitamin C; while ‘Hongyang’ balanced sugar–acid ratios with strong antioxidant enzyme activity. Similarly, Jia et al. [20] profiled three elite *A. eriantha* cultivars (MM-11, MM-13, MM-16), finding that MM-11 was richer in sugars, whereas MM-13 and MM-16 accumulated more phenolics and vitamin C, with organic acids (citric, quinic, malic, succinic) also varying. These findings illustrate how genetics shape carbohydrate metabolism, vitamin C biosynthesis, and antioxidant pathways.

Pigmentation and antioxidant profiles further diversify across cultivars. Yu et al. [19] compared red-fleshed ‘Hongyang’, yellow-fleshed ‘Jintao’, purple-fleshed *A. arguta* ‘Mini Amethyst’, and green *A. arguta* ‘Kuilv’, detecting 125 flavonoids, including nine anthocyanins, with pigmented cultivars accumulating richer and more diverse flavonoid profiles. Mao et al. [21] extended this view in *A. chinensis* ‘Jinyan’, identifying 301 flavonoids across tissues, with leaves and roots showing highest accumulation. In a follow-up, Mao et al. [35] studied fruit tissue zones in ‘Hongyang’ showing that red-flesh regions were enriched in flavonoids and phenolic acids, whereas carotenoids and organic acids partitioned differently across yellow vs. red tissue zones. These metabolite maps demonstrate that tissue-specificity adds another layer of cultivar diversity. Zhang et al. [34] showed that tetraploid red-fleshed hybrids (‘05D’ and ‘11E’) from wild x cultivated crosses accumulated significantly higher levels of flavonoids, carotenoids, and vitamin C than their diploid parents, highlighting the added complexity introduced by hybrid breeding. Similarly, Hu et al. [2] compared the fruit profiles of *A. chinensis* ‘Hongyang’ and *A. eriantha* ‘White’, found distinct patterns in aromatic amino acid metabolism: *A. eriantha* accumulated higher levels of phenylalanine- and tryptophan-derived metabolites, while *A. chinensis* produced greater amounts of serotonin and tryptamine. These differences illustrate species-specific metabolic specializations that shape both sensory attributes and nutritional quality.

Orchard management practices add another layer of metabolic heterogeneity even within a single cultivar. Rowan et al. [86] reported more variation within-vines variability, with ‘Hayward’ showing a greater percentage (90%) than ‘Zesy002’ (70%). This variability was influenced by factors such as rootstock and *Pseudomonas syringae* pv. actinidiae infection, underscoring how orchard conditions and disease pressure significantly influence metabolic expression. Taken together, these studies establish that both interspecific comparisons (*A. arguta*, *A. eriantha*, *A. chinensis*, and *A. deliciosa*) and intraspecific cultivar evaluations (‘Hongyang’, ‘Hayward’, ‘Jinyan’, ‘Kuilv’, elite *A. eriantha* lines, and hybrids) reveal distinct metabolomic signatures. Particularly, *A. eriantha* and *A. arguta* consistently emerge as metabolically superior germplasm with higher levels of vitamin C, flavonoids, and phenolics, making them valuable for breeding nutritionally enriched cultivars. At the same time, orchard heterogeneity, rootstock choice, and pathogen pressure introduce variability, emphasizing that both genetics and environment shape the metabolic foundation of kiwifruit quality.

### 4.2. Metabolite Profiling Across Development and Ripening Stages

Developmental metabolomics has provided detailed insights into the dynamic biochemical reprogramming that occurs during kiwifruit growth, maturation, and ripening. Huang et al. [84] monitored phenolic dynamics in *A. eriantha*, *A. chinensis*, and *A. deliciosa*, showing that while total phenolics declined across ripening, *A. eriantha* consistently retained markedly higher gallic acid concentrations, which correlated with the nutritional potential of wild germplasm and its superior antioxidant activity. Li et al. [85] complemented this perspective by comparing *A. arguta* ‘Kuilv’, *A. chinensis* ‘Hongyang’, and *A. deliciosa* ‘Hayward’, showing that vitamin C and sucrose accumulation in ‘Kuilv’ corresponded to its shorter development cycle, while ‘Hayward’ was dominated by glucose and fructose. In contrast, ‘Hongyang’ displayed a favorable sugar–acid balance and strong antioxidant enzyme activity, highlighting how developmental period interacts with cultivar physiology to determine harvest quality.

Temporal metabolite profiling in ‘Jinshi 1’ revealed early peaks of organic acids (citric, quinic, malic) that declined toward maturity, concurrent with rising sucrose and hexose levels, while vitamin C accumulated via the L-galactose pathway and stabilized at maturity as oxidative breakdown slowed [87]. Complementarily, Y. Xiong et al. [88] mapped color development in the same cultivar, showed that anthocyanins and most flavonoids peaked at 20 d but declined sharply by 58 d, whereas carotenoids accumulated later, with β-cryptoxanthin and β-carotene becoming the dominant pigments at maturity (175 d). Together, these studies illustrate that sugars, acids, vitamin C, and pigments follow distinct yet coordinated temporal trajectories to shape ripening.

Metabolomics studies across developmental stages have clarified the biochemical basis of kiwifruit sensory attributes by mapping in key metabolites. In ‘Hongyang’, ripening is marked by progressive accumulation of soluble sugars from starch degradation, decline in proanthocyanidins, and a marked rise in anthocyanins and vitamin C recycling [16]. Profiling of 12 ripening stages further revealed dynamic changes in sugars, organic acids, and volatiles closely aligned with ripening progression [89]. Beyond sugars and phenolics, amino acid and lipid metabolism also contribute, as shown by Tian et al. [24], who identified aspartate aminotransferase (ASP3) as central to amino acid changes during ‘Hayward’ ripening, linked with concurrent shifts in organic acids and sugars. Collectively, these studies highlight how coordinated changes in sugars, acids, vitamins, pigments, amino acids, and volatiles shape kiwifruit quality during development and ripening.

### 4.3. Metabolomic Shifts During Postharvest Storage

Postharvest metabolomics has demonstrated that storage conditions profoundly shape fruit quality by reprogramming sugars, acids, volatiles, and antioxidants. Cold storage remains the most widely applied strategy, and metabolomic evidence shows that it not only prolongs shelf life but also helps maintain nutritional quality. Choi et al. [90] profiled ‘Hayward’ and ‘Haegeum’ during four months at 0 °C, revealing progressive softening and soluble sugar accumulation in both cultivars, but with distinct metabolic patterns. ‘Haegeum’ accumulated more amino acids, fatty acids, and organic acids, whereas ‘Hayward’ showed higher sugar levels. Importantly, vitamin C and phenolics were maintained or even enhanced, and antioxidant activity (DPPH, ABTS, FRAP) was preserved, particularly in ‘Haegeum’. These findings highlight cultivar-specific metabolic shifts under low temperatures. Mao et al. [91] provided complementary insights in ‘Jinyan’, showing consistent increases in sucrose, glucose, and fructose with a decline in citric acid. These changes were coupled with depletion of respiratory intermediates from the Embden–Meyerhof–Parnas (EMP) glycolytic pathway, the tricarboxylic acid (TCA) cycle, and the pentose phosphate pathway (PPP), together with accumulation of amino acids. Intriguingly, cytokinin profiling revealed sharp increases late in ripening, suggesting possible hormone–enzyme interactions underlying postharvest softening.

Yuan et al. [92] clarified flavor development in ‘Xuxiang’, showing that volatile organic compounds (VOCs) profiles shift from aldehyde- and alcohol-dominated in early storage (0–48 h) to ester-rich later stages (after 60 h) at room temperature. These transitions aligned with firmness loss and enhanced sensory sweetness, with aldehydes correlated to respiration and tissue breakdown, while esters were positively associated with consumer appeal, defining 72–84 h as the optimal ‘ready to eat’ window. Medic et al. [93] extended these insights to kiwiberries, showing that low temperature (1 °C) best preserved firmness and storability in green cultivars (‘Jumbo’ and ‘Bingo’), whereas 6 °C enhanced anthocyanin accumulation and flavor metabolites in red-fleshed ‘Purpurna Sadowa’. Together, these findings underscore the trade-offs between storability and sensory quality, highlighting metabolomic biomarkers such as sugars, organic acids, volatiles, and anthocyanins as guides for cultivar-specific, temperature-tailored storage strategies.

### 4.4. Metabolomic Shifts During Pre- and Postharvest Treatments

Pre-and postharvest treatments profoundly influence the metabolite profile of kiwifruit, reshaping pathways that govern ripening, aroma, antioxidant properties, and shelf life. Recent metabolomics studies highlight how coatings, hormonal elicitors, physical interventions, and innovative packaging systems reprogram primary and secondary metabolism. Cao et al. [94] investigated hardy kiwifruit treated with chitosan (CTS) and chitosan–silica nanoparticle coatings (CTS-SiNPs) and showed that both treatments delayed ripening, reduced weight loss, decay, and ethylene production, while broadly downregulating volatile synthesis. Notably, CTS-SiNPs shifted the aroma profile away from consumer-preferred sweet and floral notes toward ‘herbal’ tones by elevating green-leaf aldehydes and alcohols and suppressing terpenoid biosynthesis. While such coatings prolong shelf life, their potential to alter desirable sensory traits underscores the need for integrated metabolomics-sensory validation. Other studies confirm that physical, chemical, and biological interventions significantly reshape fruit metabolism. For instance, bagging, though applied preharvest, exerts strong postharvest effects, reducing sugars, pigments, and ascorbic acid while altering phenolic, flavonoid, and antioxidant metabolism [17]. Jue et al. [25] further showed that chitosan coatings delayed softening by preserving phenolics and antioxidant metabolites, while Li et al. [26] demonstrated that 1-MCP treatments reprogrammed lipid and sugar metabolism to delay senescence. Among physical interventions, Xu et al. [27] reported that blue-light exposure maintained higher antioxidant metabolites while suppressing ethylene-related pathways, and Wang et al. [30] showed that ozone treatment preserved firmness, slowed cell wall degradation, and enhanced phenolic and antioxidant accumulation, with metabolomics corroborating shifts in phenylpropanoid and lipid pathways. In addition, Long et al. [39] introduced riboflavin-based photosensitization, demonstrating altered volatile and phenolic metabolism that improved resistance to *Botrytis cinerea*.

Hormonal elicitors and packaging further illustrate metabolomics-guided insights. MeJA alleviates chilling injury by suppressing ethylene biosynthesis, limiting cell wall degradation, and maintaining higher pools of antioxidants Niu et al. [31], with preharvest sprays proving more effective than postharvest applications by inducing stronger antioxidant and defense-related metabolite accumulation, reducing soft rot, and preserving favorable aroma profiles [40]. These findings emphasize that optimizing application timing is essential to maximize metabolic resilience. Advances in packaging, such as visible light-responsive chitosan composite films, delay quality deterioration of kiwifruit by stabilizing phospholipid metabolites and maintaining membrane integrity [29]. Collectively, these studies illustrate that pre- and postharvest interventions alter metabolomic networks across sugars, acids, volatiles, phenolics, and antioxidants in treatment- and cultivar-specific ways, with metabolomics revealing both beneficial effects and unintended consequences that guide the rational design of interventions balancing extended shelf life with consumer-preferred quality. Table 3 presents metabolomic studies in kiwifruit, highlighting cultivar-specific profiles, developmental and ripening dynamics, storage-associated metabolic shifts, and responses to postharvest treatments.

## 5. Integrated Omics Approaches in Kiwifruit

Advances in genomics, transcriptomics, proteomics, and metabolomics now allow kiwifruit biology to be examined as coordinated gene-protein-metabolite systems rather than isolated traits. High-quality genome resources and pangenome frameworks enable discovery of causal variation, while stage-specific multi-omics maps across development and storage link transcription factors, enzymes, and metabolites to sugars, organic acids, volatiles, pigments, firmness, and vitamin C dynamics. These approaches also clarify how postharvest interventions and distribution-related stresses reprogram starch and cell-wall metabolism, hormone signaling, antioxidant networks, and flavonoid pathways, while emerging transcript and metabolite biomarkers provide predictive power for maturity, postharvest quality, and storability. The sections that follow synthesize: (Section 5.1) conceptual foundations and genomic resources; (Section 5.2) development, maturity, ripening, and biomarkers; (Section 5.3) omics-informed stress and postharvest responses; and (Section 5.4) omics-driven postharvest quality, emphasizing integrated omics not as an optional addition, but as an essential frontier for precise, eco-friendly, and commercially viable postharvest management in kiwifruit.

### 5.1. Conceptual Foundations and Genomic Resources

The application of omics to postharvest management has transformed kiwifruit research from a focus on individual traits to a systems-level perspective. Earlier studies, relying primarily on physicochemical assessments or single-omics approaches, offered useful insights but provided only a partial understanding of the complex networks underpinning ripening, senescence, and stress responses. Reviews such as Belay and James Caleb [13] positioned integrated transcriptomics, proteomics, and metabolomics as transformative, emphasizing that these processes can only be fully understood by mapping coordinated changes across genes, proteins, and metabolites. Habibi et al. [14] reinforced this foundation, by demonstrating that multi-omics integration not only clarifies molecular mechanisms of quality and stress response but also identifies biomarkers that guide breeding and storage practices.

In kiwifruit specifically, the availability of high-quality genomic resources has accelerated the scope and precision of research. Nazir et al. [1] traced progress from early draft assemblies to chromosome-level references, coupled with QTL mapping and the establishment of specialized databases such as the Kiwifruit Genome Database (KGD), which has become an essential tool for modern breeding. The launch of the Kiwifruit PanGenome Database (KPGD) further represents a milestone, integrating 55 genome assemblies across 33 accessions and linking them to transcriptomic datasets, represents a further milestone by enabling discovery of structural variants and trait-associated alleles [42]. Together, these resources mark the transition from single-reference to population-scale genomic frameworks, essential for dissecting complex traits in a polyploid crop with strong genotype x environment interactions.

Proteomic and metabolomic investigations further illustrate how methodological evolution has laid the groundwork for multi-omics integration. Shin et al. [95] applied gel-based proteomics and MALDI-TOF/TOF mass spectrometry to characterize ethylene-regulated ripening in *A. deliciosa* cultivars ‘Hayward’ and ‘Garmrok’. While the study identified key proteins such as actinidain, kiwellin, and ACC oxidase, as well as cultivar-specific responses, it also highlighted the limitations of gel-based proteomics, including incomplete proteome coverage and limited functional resolution. Similarly, Yu et al. [19] combined metabolomic and transcriptomic profiling across *A. chinensis* ‘Hongyang’ and ‘Jintao’, *A. arguta* ‘Mini Amethyst’, and *A. arguta* ‘Kuilv’ cultivars, revealing diversity in flavonoid and anthocyanin accumulation and their regulation by MYB-bHLH-WD40 transcription factor complexes. This provided one of the first clear examples of gene–metabolite co-regulation in kiwifruit and underscored the importance of high-throughput platforms, multi-cultivar comparisons, and functional validation of candidate regulators.

Collectively, these advances establish the conceptual foundation of kiwifruit omics on three pillars: (i) recognition that postharvest traits are governed by gene-protein-metabolite networks rather than isolated events; (ii) establishment of robust genomic resources, from reference assemblies to pangenomes, that provide scaffolds for identifying causal variants and regulatory elements; and (iii) demonstration by early proteomic and metabolomic studies that integrative approaches can uncover nutritional and quality traits invisible to conventional analyses. These pillars now enable the application of omics to ripening regulation, stress responses, and postharvest quality in kiwifruit.

### 5.2. Fruit Development, Maturity, Ripening, and Biomarkers Across Cultivars

Kiwifruit development and ripening are complex processes shaped by coordinated transcriptional and metabolic networks, with significant variability across species and cultivars. Early transcriptomic and metabolomic profiling revealed that sugars, organic acids, amino acids, pigments, and antioxidants undergo dynamic and stage-specific reprogramming, which in turn determine flavor, texture, color, and nutritional quality [16,87,89]. Integrated omics studies now identify key regulatory mechanisms and molecular biomarkers that distinguish developmental stages, predict storage performance, and reveal cultivar-specific metabolic strategies.

Developmental stage specific multi-omics has uncovered the sequential orchestration of sugar metabolism, pigment accumulation, and vitamin C regulation during development and ripening. For example, Shu et al. [16] constructed a high-resolution gene–metabolite network in red-fleshed *A. chinensis* ‘Hongyang’, identifying transcription factors (*AcMYB123-2*, *AcERF192*) that regulate proanthocyanidin biosynthesis and anthocyanin accumulation, parallel to ascorbate recycling genes influencing vitamin C accumulation. Similarly, R. Wang et al. [89] integrated metabolomics and transcriptomics across 12 stages of ‘Hongyang’, linking starch degradation and volatile ester biosynthesis to *ERF* and *NAC* regulators. These studies demonstrate that fruit development proceeds through coordinated activation of distinct metabolic modules, regulated by transcriptional hierarchies.

Comparative omics analysis revealed strong genotype-dependent variation among cultivars and species during development. Xiong et al. [87] identified sucrose synthase (SUS) and sucrose phosphate synthase (SPS) as major contributors to sugar accumulation during later stages of development, while early activation of GDP-L-galactose phosphorylase (GGP) and GDP-L-galactose pyrophosphorylase (GPP) governed ascorbate dynamics. By contrast, Hu et al. [2] demonstrated that *A. eriantha* accumulates higher aromatic amino acids (phenylalanine, tryptophan), while *A. chinensis* preferentially channels flux toward serotonin and tryptamine, highlighting divergent nutritional signatures across species. Yu et al. [19] revealed that pigmented cultivars (‘Hongyang’, ‘Mini Amethyst’) accumulate richer and more diverse flavonoid and anthocyanin profiles than green or yellow cultivars, regulated by myeloblastosis (MYB), basic helix–loop–helix (bHLH), and tryptophan-aspartic acid 40-repeat (WD40) (MYB-bHLH-WD40) transcription factor complexes. Zhang et al. [34] further showed that tetraploid breeding lines accumulate enhanced anthocyanins, carotenoids, and vitamin C compared with diploid parents, linking ploidy manipulation to nutritional gain. These results show that developmental patterns vary among cultivars and species, and such genetic differences need to be considered when building general models of kiwifruit development and ripening.

One of the most significant translational outcomes of omics is the identification of molecular biomarkers for maturity and storability. Favre et al. [41] integrated transcriptomics, metabolomics, and enzyme assays in *A. chinensis* ‘Zesy002’, demonstrating that conventional indices (SSC, firmness, dry matter) were unreliable for discriminating early harvest stages, whereas a set of five transcript-based biomarkers (*MYB10*, *TIP4-1*, *BAM3.2*, *PMEi*, and *α-terpineol synthase*) consistently predicted storage outcomes across orchards and seasons. Similarly, Choi et al. [5] expanded the biomarker framework by linking transcriptomic shifts in cell wall-modifying enzymes (polygalacturonases, pectin methylesterases, expansins, and xyloglucan endotransglucosylase/hydrolases) and ethylene biosynthetic and signaling genes (*ACS*, *ACO*, *ETR*, *ERFs*) to ripening progression and postharvest softening. These studies also highlighted secondary metabolism genes (*PAL*, *C4H*, *4CL*, *CHS*, *CHI*, *F3H*, *DFR*, *FLS*, *UFGT*) as indicators of nutritional and sensory changes during storage. These studies suggest that molecular assays can complement traditional indices, though translation into rapid and scalable diagnostic assays remains an ongoing challenge.

Omics studies also reveal how tissue-specific expression contributes to trait formation. Mao et al. [21] profiled flavonoid distribution across roots, stems, leaves, and fruits of ‘Jinyan’, identifying tissue-enriched compounds and transcription factors linked to stress-related flavonoids. Mao et al. [35] extended this with spatial metabolomics in ‘Hongyang’, showing distinct accumulation of flavonoids, phenolic acids, and carotenoids across peel, red flesh, and core tissues, with candidate regulators (*MYB*, *bHLH*, *WRKY*, *MADS*) validated by qRT-PCR. Such studies highlight the spatial dimension of metabolite regulation, expanding our understanding beyond whole-fruit averages. Overall, the integration of transcriptomics, metabolomics, and breeding studies has advanced kiwifruit developmental biology from descriptive models to predictive frameworks. The combined evidence demonstrates that: (i) development and ripening are driven by coordinated gene–metabolite networks, (ii) species and cultivar differences shape nutrient and flavor profiles, (iii) molecular biomarkers provide improved clarity of maturity and storability compared to conventional indices, and (iv) tissue-specific analyses reveal hidden regulatory complexity. These advances provide the mechanistic foundation for translating omics into cultivar selection, harvest management, and supply-chain optimization.

### 5.3. Omics-Informed Stress and Postharvest Responses

Postharvest storage and distribution expose kiwifruit to diverse abiotic and biotic stresses, including cold and freezing injury, temperature fluctuations, hypoxic atmospheres, and pathogen attack. Integrated omics approaches have revealed that these stresses reprogram carbohydrate metabolism, hormone signaling, antioxidant defense, secondary metabolite biosynthesis, and lipid remodeling. By identifying genes, proteins, and metabolites serve as biomarkers of resistance or susceptibility, omics studies provide mechanistic insights that can guide cultivar-specific storage strategies and tailored postharvest interventions.

Comparative metabolomic and transcriptomic analysis in *Actinidia arguta* demonstrated genotype-dependent cold tolerance. The freezing-tolerant ‘Kuilv’ (KL) upregulated chalcone isomerase (CHI) and anthocyanin acyltransferase (AAT), leading to accumulation of procyanidins, quercetin, and kaempferol glycosides, whereas the sensitive ‘Ruby-3’ (RB) relied on nucleotide and phenolic acid metabolism, showing weaker dormancy and greater membrane damage [15]. Both genotypes accumulated lysophosphatidylcholines (LPCs), lysophosphatidylethanolamines (LPEs), and free fatty acids, indicating lipid degradation as a biomarker of chilling injury. A complementary multi-omics study comparing *A. chinensis* and *A. eriantha* further revealed species-specific differences in aromatic amino acid metabolism: *A. eriantha* upregulated tryptophan synthase (TSA1, TSB2), tryptophan aminotransferase (TAA), and tyrosine aminotransferase (TAT), driving phenylalanine and tryptophan accumulation, while *A. chinensis* favored serotonin/tryptamine biosynthesis [2]. These contrasting molecular signatures highlight antioxidant and amino acid pathways as key determinants of cold tolerance.

Repeated temperature fluctuations accelerated ripening and oxidative damage in *Actinidia chinensis* ‘Hongyang’. Integrated transcriptomic and metabolomic analyses revealed rapid activation of *ACS*, *ACO*, and *ETR* genes, accompanied by activation of starch and sucrose conversion enzymes including BAMs, SPS, PFK, and TPP [32]. Concurrent repression of flavonoid biosynthetic genes (*4CL*, *CHI*, and *F3′H*) reduced flavonoid accumulation. Weighted gene co-expression network analysis identified transcription factors from the *NAC*, *ERF*, *bZIP*, and *MYB* families as regulatory hubs, with *AcHsfA3a* emerging as a key regulator associated with flavonoid decline. These findings highlight the potential of *AcHsfA3a* and flavonoid pathway suppression as biomarkers for compromised cold-chain integrity.

Ethylene and its antagonist 1-MCP elicit contrasting multi-omics responses. In red-fleshed ‘Hongyang’, ethylene accelerated softening, SSC accumulation, and phenolic enrichment via induction of *AcPAL1/2*, whereas 1-MCP delayed ripening, preserved organic acids, and downregulated lipid metabolism [26]. Similarly, melatonin reshaped the transcriptomic and metabolic profile of postharvest ‘Jinyan’, upregulating ascorbate biosynthetic and recycling genes (*AcGME2*, *AcGalDH*, *AcGalLDH*, *AcDHAR*, *AcMDHAR*), suppressing degradation genes (*AcAO*), and enhancing antioxidant enzymes (SOD, CAT, APX). These omics changes stabilized ascorbic acid and phenolic content, reduced ROS-induced lipid peroxidation, and delayed senescence [66]. Nitric oxide (NO) exerted multi-level regulation: metabolically, it activated PAL, POD, chitinase, and β-1,3-glucanase, boosting phenolics, flavonoids, lignin, and HRGP to strengthen defense and vitamin C retention [63]; transcriptomically, it suppressed cell wall-degrading enzymes (PG, PL, PE, β-GAL), downregulated ethylene biosynthesis/signaling genes (*ACO*, *ERS1*, *ETR2*, *ERFs*), and repressed β-amylases while promoting cellulose synthases (*CESA1/3/6/9*) and Ca^2+^ signaling components [76]. Collectively, hormone- and signaling-based treatments converge on hormone crosstalk, redox stabilization, and secondary metabolism, offering candidate trait-associated markers that combine hormone-related transcripts with antioxidant enzyme activities.

Calcium chloride dips delayed ripening by activating Ca^2+^ signaling cascades and later modulating *AP2/ERF* and *NAC* transcription factors, altering ethylene signaling, MAPK activity, and cell wall remodeling. Proteomic and metabolomic analyses revealed changes in cysteine modifications, ubiquitination, and shifts in metabolites such as catechin and procyanidin B2 [37]. Chitosan coatings in ‘Hongyang’ suppressed ripening-associated transcripts (*AcPG1*, *AcBAM3L*, *AcBAM3.1*; *TFs AcNAC083*, *AcERF14*, *AcRAP2-10*, *AcHSFB2a*) while upregulating *F3′H* and *AcBEL1*, maintaining flavonoid compounds such as dihydrokaempferol-7-O-glucoside and eriodictyol-3′-O-glucoside [28]. These molecular signatures indicate that inhibition of starch- and cell wall-degrading enzymes combined with flavonoid enrichment are reliable biomarkers of coating efficacy.

Preharvest shading/bagging exerts lasting transcript–metabolite effects on postharvest fruit quality. In *A. eriantha* ‘Ganlv 1’, shading reduced sugars, pigments, and ascorbic acid by downregulating *DHAR*, *MDHAR*, *APX*, *GalUR*, and *GulLO*, thereby disrupting the AsA–glutathione cycle [17]. By contrast, in ‘Hongyang’, bagging increased SSC, carotenoids, and anthocyanins through repression of *GLUTR* and *LCY-ε*, coupled with upregulation of *F3M* and several *MYB/bHLH* transcription factors [25]. These results underscore how preharvest interventions modulate sugar, pigment, and antioxidant pathways with downstream effects on postharvest storability.

Although omics studies of kiwifruit–pathogen interactions remain limited, insights from other fruit crops reveal conserved defense mechanisms. Reviews by Belay and James Caleb [13] and Habibi et al. [14] summarize evidence from apple, banana, citrus, grape, litchi, mango, and tomato, where antagonistic yeasts such as *Yarrowia lipolytica* and *Wickerhamomyces anomalus* consistently trigger transcriptional and metabolic reprogramming. In apple and banana, yeast treatments enhance phenylpropanoid metabolism through induction of PAL, C4H, and 4CL. In grape and citrus, biocontrol agents stimulate antioxidant enzyme activity, notably APX, SOD, and CAT, thereby improving ROS scavenging. Similarly, in litchi, mango, and tomato, combined transcriptomic and metabolomic analyses show coordinated activation of phenylpropanoid and flavonoid pathways, together with redox-regulating enzymes, leading to reduced pathogen colonization and delayed decay. These conserved defense-associated transcriptional and metabolic profiles represent promising indicators that could be adapted and validated for biocontrol efficacy in kiwifruit.

### 5.4. Omics-Driven Postharvest Quality Management

Postharvest quality in kiwifruit is governed by an integration of physiological, biochemical, and molecular processes that determine firmness, flavor, nutritional composition, pigmentation, and storability. Integrated omics approaches have advanced beyond descriptive physiology to identify regulatory genes, metabolites, and networks that function both as mechanistic explanations and as practical biomarkers for managing quality across storage and supply chains.

Proteomic–metabolomic mapping of ripening *Actinidia deliciosa* ‘Hayward’ showed that starch breakdown is mainly mediated by β-amylases (BAMs), while sucrose and hexose accumulation involve sucrose synthases (SUS) and invertases (INV); fruit softening is driven by PG, EXP, and PME, coordinated by ethylene signaling [24]. Complementary evidence from calcium dips showed that Ca^2+^ reduced ethylene biosynthesis and delayed softening through regulation of *AP2/ERF* and *NAC* transcription factors, while associated shifts in catechin and procyanidin B2 profiles highlighted its dual structural and signaling roles [37]. Similarly, biochemical elicitors such as γ-aminobutyric acid (GABA) suppress starch breakdown and cell wall disassembly by downregulating BAMs, PGs, and expansins, thereby maintaining firmness [67]. Together, these omics-informed insights highlight how metabolic and regulatory networks can be targeted to design interventions that preserve firmness and textural quality during kiwifruit storage.

Multi-stage metabolomic–transcriptomic profiling of *A. chinensis* ‘Hongyang’ clarified sugar and acid metabolism, with INV, SPS, and BAM orchestrating sugar accumulation, while DAHPS and QDH regulate organic acids. Aroma volatile production was linked to transcription factors *AcERF182* and *AcNAC4*, validated through promoter activation assays as regulators of ester biosynthesis [89]. These molecular insights not only clarify sensory drivers but also present direct targets for breeding cultivars with optimized flavor and for postharvest interventions that sustain consumer acceptability.

Gene–metabolite networks similarly regulate postharvest pigmentation and antioxidant dynamics. In *A. arguta* ‘Jinhongguan’, anthocyanin accumulation during storage was dominated by cyanidin-3-O-galactoside, under the regulation of phenylpropanoid and flavonoid genes (*AaPAL3*, *Aa4CL3*, *AaCHS2/3/8/9/11*, *AaDFR1/2*, *AaANR1*, *UFGT3a*, and *UFGT6b*) and TFs *MYB108* and *bHLH30* [18]. In *A. chinensis* ‘Jinshi 1’, carotenoid biosynthesis (β-carotene, β-cryptoxanthin) was directed by MYB-bHLH complexes [88]. These findings emphasize that pigment and antioxidant pathways remain transcriptionally active postharvest, shaping both nutritional and visual quality.

Omics studies have clarified the molecular basis of several postharvest treatments in kiwifruit. Atmospheric ozone delayed softening by downregulating cell wall-modifying enzymes such as PG, PME, and XTH while sustaining cellulose synthase, and complementary metabolomic data show elevated phenolic content and enhanced antioxidant enzyme activity (SOD and CAT), underscoring its dual role in structural preservation and oxidative stress mitigation [30,96]. Hormonal and signaling regulators also play pivotal roles: MeJA mitigates chilling injury by suppressing ethylene biosynthesis, stress-related transcription factors, and cell wall hydrolases, while sustaining antioxidant defenses [31]. Melatonin (MT) contributes to redox homeostasis and nutritional stability by upregulating vitamin C biosynthetic and recycling genes (*AcGME2* and *AcMDHAR1*) while repressing degradation genes such as *AcAO*, thereby maintaining AsA pools and delaying senescence [66]. Physical cues like blue light further reprogram ripening, reducing ethylene production and suppressing the expression of starch- and cell wall-related genes in ‘Jinyan’, which translates into delayed softening and extended shelf life [27]. Building on these single-signal strategies, multi-hormone treatments such as a mixture of brassinolide, melatonin, MeJA, and salicylic acid act synergistically, preserving firmness, vitamin C, and organic acids while strongly suppressing ethylene biosynthesis, outperforming individual applications [68]. By contrast, CPPU enhances fruit size but introduces significant trade-offs, as its effects on storability and flavor are highly dose- and temperature-dependent, altering sugar–acid balance, tannin profiles, and antioxidant levels [69]. Together, these omics-informed insights demonstrate that interventions spanning atmospheric (ozone), hormonal (MeJA, melatonin, multi-hormone), and physical (blue light) domains converge on common pathways of ethylene regulation, antioxidant metabolism, and cell wall dynamics, providing a mechanistic foundation for precision strategies to extend shelf life while safeguarding the sensory and nutritional quality of kiwifruit.

Traditional maturity and quality indices such as SSC and firmness often fail to reliably predict storage performance. Transcriptomic profiling of early-harvested *A. chinensis* ‘Zesy002’ identified five biomarkers (MYB10, TIP4-1, BAM3.2, PMEi, and α-terpineol synthase) that effectively differentiated maturity stages and predicted the risk of storage breakdown across orchards and seasons [41]. When integrated with SSC, these biomarkers improved accuracy in predicting storability, highlighting their practical application for postharvest management.

In summary, Table 4 compiles integrated omics studies in kiwifruit, outlining major research focuses, key discoveries, and their physiological and postharvest implications. Despite their promise, omics-based biomarkers are inherently destructive and laboratory-intensive, which limits their direct industry application. Nondestructive technologies (spectroscopy, hyperspectral imaging, acoustic profiling, chemometric models) provide a complementary solution. Once calibrated with omics datasets, these platforms can translate transcriptomic or metabolomic predictive markers into rapid, field- or packhouse-ready tools. For instance, markers of firmness (BAM3.2, regulating starch degradation; PMEi, modulating cell wall disassembly), storability (MYB10, influencing flavonoid biosynthesis; TIP4-1, linked to membrane integrity), and pigmentation (anthocyanin-regulating MYBs controlling cyanidin accumulation) correspond directly to key physiological traits such as texture, shelf life, and flesh coloration. Integrated with nondestructive platforms, these predictive markers enable real-time, non-invasive classification of maturity and quality, marking the emerging frontier of precision postharvest quality management in kiwifruit.

## 6. Nondestructive Quality Estimation in Kiwifruit

The increasing demand for rapid, objective, and non-invasive quality evaluation has accelerated the adoption of nondestructive estimation in horticultural crops, with kiwifruit emerging as a model crop. Traditional destructive methods are reliable but inefficient for commercial practice, whereas recent advances in spectroscopy, imaging, and machine learning (ML) provide robust alternatives for the development of rapid, low-cost, reliable and reproducible nondestructive alternatives. Comprehensive reviews on nondestructive technologies emphasize the transformative role of hyperspectral imaging (HSI), visible and near-infrared (VNIR) spectroscopy, and other sensor modalities in fruit research, while also underscoring persistent challenges such as high costs, variability across environments, and limited robustness under commercial conditions [98,99]. Within kiwifruit, research spans a broad spectrum of applications, from predicting internal quality attributes to detecting hidden defects, evaluating chilling injury and fungal infections, and forecasting storability and shelf life.

### 6.1. Target Quality Traits

Prediction of SSC, dry matter (DM), pH, and firmness remains central to nondestructive kiwifruit research. Various studies have demonstrated the strong potential of HSI across VNIR and NIR ranges. J. Li et al. [100] applied NIR-HSI (1000–2500 nm) to 324 ‘Fenghuang-1’ fruits and achieved strong prediction for textural properties (r > 0.9), though shear-related properties were less reliable due to methodological errors in shear testing. Similarly, Shang et al. [101] applied VNIR HSI to ‘Guichang’ fruit and reported high predictive performance for SSC and firmness, with competitive adaptive reweighted sampling (CARS)-based models achieving R^2^p values of 0.896 and 0.871, respectively, while color traits were less robust. X. Wang et al. [102] compared fluorescence spectral imaging (FSI, 400–1000 nm) with HSI (387–1034 nm) in ‘Hongyang’, showing that FSI outperformed HSI for SSC prediction, with extreme learning machine models reaching R^2^p = 0.889.

Beyond HSI, alternative spectroscopic and structural analyses have been explored. Tian et al. [103] used bulk optical properties derived from integrating-sphere measurements and demonstrated that absorption coefficients correlate strongly with SSC, while scattering coefficients align with firmness, outperforming conventional VNIR transmission spectra. Berardinelli et al. [104] introduced a microwave waveguide system for low-cost firmness prediction, while acoustic and vibration approaches continue to show promise. W. Zhang et al. [105] applied an impact vibration method with back-propagation neural networks, achieving about 92% accuracy for prediction of firmness and sensory quality. Zhang et al. [106] advanced this further by coupling acoustic elasticity indices with kinetic modeling, providing accurate shelf life predictions across temperature ranges. More recently, Nouri and Abdanan Mehdizadeh [107] developed a vibration-based firmness device using random forest regression, achieving R^2^ = 0.96, while Sneddon et al. [108] validated acoustic stiffness and compression methods on over 4000 ‘SunGold’ fruits, confirming strong within-season correlations but reduced robustness across years. Collectively, these works illustrate diverse spectral and mechanical options for nondestructive firmness assessment.

Classifying maturity and ripeness represents another key application. Benelli et al. [109] used HSI (400–1000 nm) to predict SSC and firmness in ‘Hayward’, achieving R^2^ values above 0.9 and classifying fruit into unripe, storage-ripe, and consumer-ripe categories with robust discrimination of sensitivities exceeding 90%. Shang et al. [101] achieved 98.3% accuracy for maturity classification using simplified k-nearest neighbor models, while Ma et al. [110] introduced an object-rotation NIR-HSI system, generating 360° maps of SSC and pH gradients across golden kiwifruit. Meng et al. [111] further provided visualization maps linking SSC and firmness to ripening progression. Compact and affordable systems are also emerging: Yang et al. [43] developed a LED–photodiode device capable of classifying sweetness and firmness in under three seconds with 85–91% accuracy, and Li et al. [44] integrated smartphone-based RGB chroma indices with GC-MS volatile profiling to determine freshness windows in ‘Xuxiang’ fruits. Together, these studies illustrate the transition from laboratory to practical tools for growers, suppliers, and consumers.

Nondestructive techniques also enable early detection of hidden bruises and physiological disorders. Ebrahimi et al. [112] combined HSI with 2D and 3D CNNs to detect bruises in ‘Hayward’, achieving up to 98–100% accuracy in unripe fruit, though detection was less reliable once fruit ripened. Liang et al. [113] demonstrated that structured HSI provides superior performance compared with conventional HSI, achieving 100% accuracy in detecting early bruises (0–4 days after impact) and correlated strongly with biochemical markers such as chlorophyll degradation and lipid peroxidation. Similarly, Haghbin et al. [114] applied HSI to identify *Botrytis cinerea* (gray mold) infections in ‘Hayward’ before the appearance of visible symptoms, achieving 96% classification accuracy through wavelength selection and linear discriminant analysis (LDA) modeling.

Chilling injury (CI) detection has also advanced through optical and structural imaging. Z. Wang et al. [115] applied VNIR interactance spectroscopy to distinguish sound and severely injured ‘Zesy002’ fruit; while Wang et al. [116] used a dual-laser system (730 and 850 nm) to classify CI severity with 85–94% accuracy. Z. Yang et al. [117] applied laser-backscattering imaging to detect CI in ‘SunGold’ and ‘Hayward’, showing higher accuracy in ‘SunGold’ due to reduced trichome interference. More advanced structural imaging by J. Wang et al. [118] used X-ray computed tomography with machine learning to classify healthy, bruised, and lignified fruit, achieving mean area under the receiver operating characteristic curve (AUC) values above 0.9 and revealing new insights into chilling-induced lignification.

Volatile- and gas-based sensing further complement physical methods in defect detection. Bakhshipour [50] showed that e-nose systems outperform HSI in classifying ripeness, and their integration further improved predictions of firmness, SSC, and titratable acidity (TA), with accuracies above 94%. Guo et al. [119] developed a flexible ethylene sensor capable of wireless data transmission and 97.5% ripeness classification accuracy. These approaches highlight the potential of combining chemical and physical sensing for improved robustness in commercial settings.

Prediction of storage performance has significant implications for reducing postharvest loss. M. Li et al. [120] tested VNIR spectra at harvest to classify fruit into “good” and “soft” categories after long storage, achieving partial success but highlighting the difficulty of predicting intermediate storability classes. Ding et al. [121] showed that FT-NIR spectroscopy outperformed a commercial firmness meter (Kiwifirm™), particularly in the critical consumer-acceptable range (10–40 N). Kinetic modeling has further extended the predictive capabilities: Zhang et al. [106] applied Arrhenius-based acoustic models to estimate shelf life across storage temperatures, while Niu et al. [122] integrated chemical kinetics and microbial dynamics into shelf life models for ‘Xuxiang’, supported by a Python (version 3.12.5) tool and QR code access for real-time predictions.

Scalability under real-world conditions is now being tested. Wang et al. [46] optimized YOLOv5 networks to achieve 98% defect detection accuracy under commercial grading conditions, while Wang et al. [45] combined ResNet34 with a convolutional block attention module (CBAM) to reach 99.6% accuracy in external defect classification. Yang et al. [47] developed an intelligent kiwifruit sorting system using impact force signals and CNNs, where the proposed peak image (PI) method achieved 98.89% accuracy while minimizing fruit damage. Portable tools such as smartphones and compact NIR sensors [43,44] are further lowering adoption barriers. Nonetheless, most approaches remain limited by single-cultivar validation, small datasets, and laboratory dependence, underscoring the need for large-scale, multi-orchard and multi-season validation for practical application.

### 6.2. Integration with Omics and Data Fusion Across Instruments

The most significant advances in nondestructive quality evaluation of kiwifruit emerge when sensing technologies are integrated with mechanistic models or omics-based frameworks, thereby linking physiological understanding with predictive accuracy. Xiao and Li [123] showed that combining NIR spectra with a physics-based softening model not only improved predictive stability but also provided an interpretable basis for linking optical data to underlying biochemical processes, outperforming conventional black-box approaches such as PLSR and SVR. Similarly, Valasiadis et al. [124] applied a spatiotemporal multi-omics framework to demonstrate how dry matter content and tissue-specific transcriptomic regulation influence starch metabolism, aquaporin-mediated water transport, and phenolic biosynthesis. These underlying biochemical and molecular processes translate into measurable sensor traits such as firmness, sweetness, and antioxidant levels, underscoring the potential of omics-informed prediction to enhance nondestructive assessments. Beyond biological integration, multimodal data fusion across instruments has also strengthened prediction robustness. Cevoli et al. [48] combined VNIR hyperspectral imaging with FT-NIR spectroscopy to exploit complementary spectral regions, achieving up to a 16% reduction in prediction error compared with single-instrument models. Likewise, Zou et al. [49] demonstrated that integrating fluorescence-HSI with advanced machine learning improved maturity classification by capturing fluorescence signals linked to chlorophyll and carotenoid degradation. In another application, Bakhshipour [50] showed that combining hyperspectral imaging with volatile analysis via an electronic nose significantly increased accuracy in predicting ripeness and quality traits, highlighting the value of multimodal sensing. Collectively, these studies illustrate how integrating spectral data, omics insights, and complementary sensing modalities moves nondestructive evaluation beyond stand-alone tools toward holistic, biologically informed, and commercially applicable systems for kiwifruit quality management. Summary of recent advances in nondestructive and data-driven technologies for kiwifruit quality evaluation and prediction is presented in Table 5.

## 7. Summary and Future Perspectives

Postharvest research in kiwifruit (*Actinidia* spp.) has advanced from descriptive physiology toward mechanistic frameworks enabled by omics and nondestructive technologies. Conventional management strategies such as preharvest treatment, cold storage, controlled atmosphere storage and packaging, treatment of ripening regulators, and coatings remain central but cannot fully explain or manage the complexity of ripening, chilling injury, lignification, or pathogen susceptibility. Transcriptomics and metabolomics have mapped gene–metabolite networks governing softening, sugar–acid balance, pigmentation, antioxidants, and stress tolerance, while integrated multi-omics has identified biomarkers predictive of maturity, postharvest quality and storability. These findings underscore the importance of species- and cultivar- specific approaches, reflecting the genetic and metabolic diversity across *Actinidia*.

Despite these advances, proteomics and lipidomics are comparatively underexplored in kiwifruit, limiting full systems-level integration. Pathogen-focused omics and microbiome interactions are underexplored, constraining development of sustainable biocontrol strategies. Most biomarker discoveries are correlative and restricted to single cultivars or storage conditions, with limited validation across seasons and environments. Current biomarkers should be viewed as putative indicators until validated with broader datasets. Furthermore, translation of omics discoveries into simple, rapid, and affordable assays for commercial use remains a critical challenge.

Future research should therefore focus on (i) expanding multi-omics datasets across diverse germplasm, growing regions, and storage systems to capture genotype × environment × management interactions; (ii) advancing proteomic, lipidomic, and epigenomic approaches to complement transcriptomics and metabolomics; (iii) integrating microbiome dynamics with pathogen-responsive omics, using high-throughput sequencing to clarify community shifts during storage and their impact on decay and quality (iv) developing simplified molecular assays or calibrating omics biomarkers with nondestructive sensing tools; and (v) embedding these advances into digital agriculture frameworks for predictive modeling and decision support. Although biomarker discovery is progressing rapidly, translating these findings into commercial diagnostic tools will require coordinated efforts among researchers, sensor developers, and industry partners as technical and economic data become available.

Overall, the convergence of omics-informed insights with nondestructive sensing technologies provides a pathway toward precision postharvest quality management, enabling sustainable production, reduced losses, and consistent nutritional and sensory quality for global consumers.

## Figures and Tables

**Table 1:** Summary of recent advances in kiwifruit postharvest physiology and technology, highlighting treatments, key outcomes, and mechanistic insights.

Factor/Treatment	Cultivar(s)	Key Findings	Mechanistic Insights	Reference(s)
Agro-ecology from 5 Turkish regions	‘Hayward’	Clear regional differences: Yalova fruits had highest Vit C and antioxidant activity; Giresun fruits maintained firmness	Environment influences antioxidant properties, phenolics, and firmness	Ozturk et al. [7]
Preharvest chitosan	‘Garmrok’	Increased storability, delayed softening, and reduced ethylene/respiration	Downregulation of *ACS/ACO* and cell-wall genes; upregulation of lignin metabolism	Kumarihami et al. [4]
Preharvest ML	‘Guichang’	Enhanced soft rot resistance, improved firmness and antioxidants	Suppressed respiration and moderated sugar accumulation; antifungal effect against *B. dothidea*	Peng et al. [9]
Preharvest MeJA	‘Guichang’	More effective than postharvest: maintained firmness, suppressed soft rot, preserved volatiles	Enhanced ROS balance, AsA-GSH cycle, defense enzyme activity	Yang et al. [32]
Harvest stage based on days after full blossom (DAFB)(160–180)	‘Hayward’, ‘Haegeum’, ‘Hongyang’	Early harvest (160 DAFB) optimized storability, but cultivar differences in ripening quality	Firmness loss linked to PG activity and pectin solubilization; respiration/ethylene drive faster softening	Choi et al. [5]
Harvest stage based on SSC (4.5–9.5%)	‘Hongyang’	Stage II (6.5–7.5% SSC) gave best chilling tolerance and balance of storability/decay	Higher antioxidant and energy metabolism enzymes sustain ATP and ROS homeostasis	Wang et al. [6]
Reappraisal of SSC index	‘Hayward’	6.2% SSC is not universal; physiological maturity varies with orchard, climate, and starch phase	Carbohydrate import and starch degradation drives SSC	Burdon et al. [8]
Mechanical stress (compression)	‘Hayward’	Compression accelerated ripening; altered cell wall components and metabolites	*AP2/ERF*, *WRKY*, *bZIP TFs* regulate stress-induced pathways; sucrose metabolism and TCA intermediates shift	Polychroniadou et al. [55]
Mechanical damage (puncture)	‘Jinli’	Equator region most vulnerable; damage accelerated respiration and ripening	Stress concentrated at equator; cell ultrastructure differences explain variation	Chen et al. [56]
Pre-cooling methods	‘Hongyang’	Forced-air cooling best preserved firmness and flavor balance; reduced oxidative stress	Forced-air cooling minimized water loss and MDA; maintained CAT activity; predictive firmness modeling validated	Zhang et al. [58]
Carotenoid dynamics at 20 and 4 °C storage	‘Jinshi 1’, ‘Jinyan’	Carotenoid levels differ by cultivar and storage temperature	*PSY*, *LCYB*, *NCED* regulation of carotenoid biosynthesis/degradation	Xia et al. [65]
Storage at 10 °C	‘Cuixiang’	Caused starchy taste unless transferred at 110 N firmness to room temperature (RT)	Incomplete starch-to-sugar conversion at 10 °C; transfer improves SSC	Chai et al. [54]
MeJA postharvest	‘Hongyang’	Reduced soft rot; maintained firmness, Vit C, and phenolics	Enhanced APX/SOD/CAT, phenylpropanoid pathway; activated defense-related genes	Xie et al. [52]
1-MCP	‘Miliang No. 1’, ‘Xuxiang’, ‘Hayward’, ‘Huayou’	Slowed ripening, reduced softening, cultivar- and concentration-dependent effects	Maintained ATP/energy charge; altered carbohydrate, amino acid, fatty acid metabolism	Huang et al. [10] Zhao et al. [60]
1-MCP and MeJA/MeSA	‘Xuxiang’	1-MCP preserved texture but aggravated lignification; MeJA/MeSA reduced lignin	*PAL*, *CAD*, *POD* upregulated by 1-MCP; suppressed by MeJA/MeSA	Li et al. [51]
1-MCP	‘Longcheng No. 2’	Delayed softening, preserved TA, Vit C, phenolics, flavonoids	Suppressed PE, PG, β-Gal, AMY, SPS, SS; maintained sucrose	Xiong et al. [23]
1-MCP and ripening	‘Guichang’	Preserved firmness, sugars, acids, antioxidants; reduced soft rot	Delayed starch degradation, stabilized antioxidant and defense enzymes	Ma et al. [11]
NO	‘Bruno’	Reduced decay, maintained Vit C, and delayed ripening	Upregulated *PAL*, *POD*, *β-1,3-glucanase*; increased phenolics, flavonoids, lignin	Zheng et al. [63]
MT postharvest	‘Bruno’, ‘Jinyan’	Reduced ethanol fermentation, delayed ripening; maintained AsA	Suppressed *PDC*/*ADH*; enhanced AsA biosynthesis/recycling genes	Cheng et al. [53] Luo et al. [66]
Cis-3-hexenyl butyrate	‘BAG1’	Accelerated ripening, enhanced anthocyanins and SSC	NIR spectroscopy tracked anthocyanins; but HB shortened storage life	Lembo et al. [57]
TeA	‘Hongyang’	Delayed softening and oxidative stress under ambient storage	Suppressed β-amylase, PL, cellulase; enhanced SOD/POD/AsA	Yan et al. [61]
H_2_O_2_	‘Hongshi’	Accelerated softening and flavor development but reduced storability	Activated starch/pectin enzymes; elevated ROS, oxidative stress	Yan et al. [62]
High O_2_/N_2_ CA	‘Hayward’	Accelerated ripening to edible firmness without quality loss	Enhanced respiration, ethylene, ATP, volatiles; upregulated ripening genes	Chai et al. [59]
Ethanol vapor	‘Huan Optimal No. 1’	Reduced chilling injury, decay, extended consumption window	Enhanced antioxidant enzymes (CAT, SOD, GR); preserved firmness and color	Xiong et al. [38]
Short-term anaerobic	‘Bingo’	24 h N_2_ treatment delayed ripening, reduced decay, enhanced antioxidants	Increased SOD, CAT, APX, phenolics, flavonoids, AsA; reduced ROS	Ying et al. [33]
Riboflavin and light	‘Hongyang’	Enhanced defense vs. gray mold; delayed softening	JA induction, sucrose/glucose balance, ROS scavenging	Long et al. [39]
Blue light	‘Jinyan’	Delayed ripening, starch degradation, and cell wall metabolism	Suppressed *ACS*/*ACO*, *BAMs*, *PG*/*PME*/*PL*; and shifts *MYB* and *bHLH*	Xu et al. [27]
GABA	‘Hongyang’	Maintained firmness, delayed starch breakdown and softening	Downregulation of *BAM*s, *PG*, *PE*, *XTH*; preserved cell wall polysaccharides	Yan et al. [67]
Phytohormone synergistic treatment	‘Yan Nong 3’	Combined Brassinolide, melatonin, methyl jasmonate and salicylic acid reduced decay, weight loss, and preserved nutrients	Suppressed ethylene biosynthesis genes; sustained acids/amino acids	He et al. [68]
CPPU and storage temperature	‘Xuxiang’	Dose- and temp-dependent: improved size, but high concentration reduced storability	Modified sugar/acid metabolism, antioxidants, pigments	Qi et al. [69]
AOS	‘Longcheng No. 2’	Improved firmness, antioxidants, suppressed chilling injury	Enhanced antioxidant enzymes; lowered respiration, MDA	Xiong et al. [38]
*Lactiplantibacillus pentosus* CW5	‘Jinyang’	Slowed softening, reduced decay, preserved Vit C	Sustained SOD, POD, APX; shifted microbiome toward beneficial bacteria	Hao et al. [64]

Acronyms: 1-MCP = 1-Methylcyclopropene; ACS = 1-Aminocyclopropane-1-Carboxylate Synthase; ACO = 1-Aminocyclopropane-1-Carboxylate Oxidase; AOS = Alginate Oligosaccharide; AP2/ERF = APETALA2/Ethylene Response Factor; APX = Ascorbate Peroxidase; AsA = Ascorbic Acid; AsA–GSH = Ascorbate–Glutathione; BAM = β-Amylase; BGA = β-Galactosidase; BGLU = β-Glucosidase; BXL = β-D-Xylosidase; CAD = Cinnamyl Alcohol Dehydrogenase; CAT = Catalase; CA = Controlled Atmosphere; CESA = Cellulose Synthase A; CPPU = Forchlorfenuron (N-(2-chloro-4-pyridyl)-N′-phenylurea); EG = Endoglucanase; EIN2L = Ethylene-Insensitive Protein 2-Like; EXP = Expansin; FAD = Fatty Acid Desaturase; GR = Glutathione Reductase; HB = Cis-3-Hexenyl Butyrate; H_2_O_2_ = Hydrogen Peroxide; JA = Jasmonic Acid; LCYB = Lycopene β-Cyclase; MAP = Modified Atmosphere Packaging; MDA = Malondialdehyde; MeJA = Methyl Jasmonate; MeSA = Methyl Salicylate; MT = Melatonin; NCED = 9-Cis-Epoxycarotenoid Dioxygenase; NO = Nitric Oxide; PAL = Phenylalanine Ammonia-Lyase; PAE = Pectin Acetylesterase; PDC = Pyruvate Decarboxylase; PE = Pectinesterase; PG = Polygalacturonase; PL = Pectate Lyase; PLIP = Phospholipase A1; POD = Peroxidase; PSY = Phytoene Synthase; ROS = Reactive Oxygen Species; RT = Room Temperature; SOD = Superoxide Dismutase; SSC = Soluble Solids Content; SS = Sucrose Synthase; SPS = Sucrose Phosphate Synthase; TA = Titratable Acidity; TCA = Tricarboxylic Acid; TeA = Tenuazonic Acid.

**Table 2:** Summary of transcriptomic studies in kiwifruit linking cultivar variation, ripening stages, postharvest treatments, and storage responses to functional genes regulating physicochemical and antioxidant traits.

Theme	Trait/Focus	Key Genes/Regulators	Cultivars	Key Findings	References
Cultivar responses	Temperature and ethylene-driven ripening	*AcACO3*, *AcXET2*, *AcPG*, *AcEXP1*, *AcPMEi*, *AcMADS2*, *AcNAC5*, *AcbZIP2*	‘Sanuki Gold’ ‘Hayward’	‘Sanuki Gold’ ripens faster at mild temperature (15 °C); ‘Hayward’ requires colder storage; transcriptomic induction differs by cultivar.	Mitalo et al. [70]
	Ethylene treatment	Cell wall genes, *ACS*, *ACOs*, *ERFs*; defense-related genes	‘Hayward’ ‘Haegeum’	Ethylene induces overlapping DEGs but with cultivar-specific stress/defense activation.	Choi et al. [71]
	Pigmentation differences	*CHYB1*, *NCED1*, *CCD1*, *CCD4*	‘Jinshi 1’, ‘Hort16A’	‘Jinshi 1’ brighter yellow due to β-cryptoxanthin; ‘Hort16A’ shows higher carotenoid degradation.	Xia et al. [72]
	Anthocyanin regulation	*AcLDOX2*, *Ac5GGT1*, *Ac5AT2*; *AcbHLH74-2 (activator)*, *AcMYB4-1 (repressor)*	‘Donghong’	Stage-specific anthocyanin accumulation regulated by MYB and bHLH TFs.	Liang et al. [22]
	Storage off-flavor	*β-amylases*, *sucrose synthases*, *PDC*, *ADH*	‘Bruno’, ‘White’	‘Bruno’ accumulates ethanol via strong fermentation-related induction; ‘White’ avoids off-flavor.	Huan et al. [77]
Ripening stages	Anthocyanins/phenolics	*AcLDOX2*, *Ac5GGT1*, *Ac5AT2*; MYB & bHLH regulators	‘Donghong’	Dynamic decline of phenolics, stage-specific anthocyanin peaks.	Liang et al. [22]
	Firmness & metabolic shifts	Cell wall genes (*PE*, *CEL*, *EXP*), organic acid/sugar metabolism genes	‘Xuxiang’	~2000 DEGs induced by ethylene; firmness is dominant driver of transcriptome shifts.	Zhang et al. [73]
	Hormonal reprogramming	ABA, CK, ethylene-related genes; *PG*, *PME*, *XTH*, *EXP*	‘Jinyan’	Dual model: T6P–SnRK1–TOR for starch metabolism; ABA–CK–ethylene for cell wall disassembly.	Lin et al. [74]
Postharvest treatments	Ethylene–auxin crosstalk	*AcGH3.1*; *AcERF1B*, *AcERF073*	‘Hayward’	Ethylene accelerates auxin degradation via ERF activation of *AcGH3.1*.	Gan et al. [75]
	Ethylene-induced ripening	Cell wall & sugar genes, *ACS*, *ACO*, *ERFs*	‘Hayward’, ‘Haegeum’, ‘Xuxiang’	Ethylene broadly induces DEGs for softening, sugar metabolism, and stress responses.	Choi et al. [71] Zhang et al. [73]
	NO	*PG, PL*, *PE*, *β-GAL*, *ACO*, *ERFs*; *CESA1/3/6/9*	‘Jinkui’	NO suppresses ethylene & cell wall genes, delays softening, and upregulates cellulose synthases.	Yang et al. [76]
Storage responses	Alcoholic off-flavor	*β-amylases*, *SS*, *PDC*, *ADH*	‘Bruno’ vs. ‘White’	‘Bruno’ prone to ethanol accumulation; ‘White’ resistant.	Huan et al. [77]
	Degreening/color loss	*SGR2*, *PAO1*	‘Zesy003’, ‘Zesh004’, ‘Hayward’	Faster de-greening in yellow cultivars, low temperatures suppress chlorophyll breakdown.	Gambi et al. [78]
	Cold tolerance	*TPS5*, *BAM3.1*, *CBF3*, *MYC2*, *MYB44*	‘KL’, ‘RB’	Tolerant genotype activates sugar metabolism and stress TFs; sensitivity does not.	Sun et al. [15]
	Starch-to-sugar conversion	*AcBAM5*, *AcBAM13*	‘Hongyang’	β-amylases regulate starch degradation; silencing increases starch.	Gong et al. [79]
	Sucrose metabolism	102 *INV* genes; ERF & bHLH regulators	‘Kukuwa’, ‘Qinhuang’, ‘Xianziguang’	INV family controls sugar accumulation; strong TF regulation identified.	Qiang et al. [80]
Physicochemical traits related genes	Softening/firmness	*AcKUP2* (K^+^ transporter, ERF-regulated)	‘Hongyang’	Links K^+^ uptake to sugar metabolism and softening.	Shan et al. [81]
	Starch degradation	*AcBAM5*, *AcBAM13*	‘Hongyang’	ABA/cold-induced; regulate sweetness and softening.	Gong et al. [79]
	Sugar accumulation	102 *INV* genes; ERFs, bHLHs	*A. arguta*	INV activity controls ripening-stage sugar profiles.	Qiang et al. [80]
Antioxidant traits related genes	Ester biosynthesis (aroma)	*AdFAD1*, *AdALDH2*, *AdAT17*; *AdNAC5 (activator)*, *AdDof4 (repressor)*	‘Hayward’	Ethylene promotes ester production; ERFs regulate aroma genes.	Zhang et al. [82]
	Anthocyanin biosynthesis	*AcLDOX2*, *Ac5GGT1*, *Ac5AT2*; *AcbHLH74-2* (activator), *AcMYB4-1* (repressor)	‘Donghong’	Stage-specific anthocyanin accumulation driven by TFs.	Liang et al. [22]
	Carotenoid biosynthesis	*CHYB1*; *CCD1*, *CCD4*	‘Jinshi 1’, ‘Hort16A’	β-cryptoxanthin in ‘Jinshi 1’ drives bright yellow color; higher carotenoid degradation in ‘Hort16A’.	Xia et al. [72]

Acronyms: ABA = Abscisic Acid; ABP = Auxin-Binding Protein; ACO = 1-Aminocyclopropane-1-Carboxylate Oxidase; ACS = 1-Aminocyclopropane-1-Carboxylate Synthase; ADH = Alcohol Dehydrogenase; ALDH = Aldehyde Dehydrogenase; BAM = β-Amylase; bHLH = Basic Helix–Loop–Helix; bZIP = Basic Leucine Zipper; CAD = Cinnamyl Alcohol Dehydrogenase; CBF = C-Repeat Binding Factor; CCD = Carotenoid Cleavage Dioxygenase; CEL = Cellulase; CESA = Cellulose Synthase; CHYB = β-Carotene Hydroxylase; CK = Cytokinin; DEG = Differentially Expressed Gene; ERF = Ethylene Response Factor; EXP = Expansin; FAD = Fatty Acid Desaturase; GH3 = Gretchen Hagen 3; INV = Invertase; KUP = Potassium Transporter; MADS = MCM1–AG–DEFICIENS–SRF Transcription Factor; MYB = Myeloblastosis Transcription Factor; NAC = NAM–ATAF–CUC Transcription Factor; NCED = 9-Cis-Epoxycarotenoid Dioxygenase; NO = Nitric Oxide; PAO = Pheophorbide a Oxygenase; PDC = Pyruvate Decarboxylase; PE = Pectinesterase; PG = Polygalacturonase; PL = Pectate Lyase; PME = Pectin Methylesterase; PMEi = Pectin Methylesterase Inhibitor; PR = Pathogenesis-Related Protein; SGR = Stay-Green Gene; SnRK1 = Sucrose Non-Fermenting-1 Related Protein Kinase 1; SS = Sucrose Synthase; TF = Transcription Factor; T6P = Trehalose-6-Phosphate; TOR = Target of Rapamycin; TPS = Trehalose–Phosphate Synthase; XET = Xyloglucan Endotransglycosylase; XTH = Xyloglucan Endotransglucosylase/Hydrolase.

**Table 3:** Summary of metabolomic studies in kiwifruit emphasizing cultivar-specific profiles, developmental and ripening trends, storage-associated metabolic shifts, and responses to postharvest treatments.

Theme	Focus/Trait	Key Metabolites	Cultivars	Main Findings	References
Cultivar comparisons	Nutritional and bioactive composition	Polyphenols, flavonoids, tannins, vitamin C, lutein, zeaxanthin, fiber	‘M1’, ‘Hayward’, ‘Bidan’	*A. arguta* richer in phenolics and vitamin C than ‘Hayward’, though lower than ‘Bidan’; stronger antioxidant activity in hardy kiwi and *A. eriantha*.	Leontowicz et al. [83]
	Growth and nutritional metabolism	Sucrose, glucose, fructose, vitamin C, antioxidant enzymes	‘Kuilv’, ‘Hongyang’, ‘Hayward’	‘Kuilv’ shortest development, highest sucrose and vitamin C; ‘Hayward’ largest fruit, higher glucose/fructose; ‘Hongyang’ intermediate with favorable sugar–acid ratio and SOD activity.	Li et al. [85]
	Orchard metabolite variability	Soluble sugars, phytohormones	‘Hayward’, ‘Zesy002’	Within-vine metabolite variability exceeded between-vine variability; influenced by infection, rootstock, environment; careful replication critical.	Rowan et al. [86]
Development and ripening	Phenolic dynamics	Gallic acid, catechin, epicatechin, rutin, caffeic acid, ferulic acid, quercetin	‘Ganmi No. 6’, ‘Hongyang’, ‘Jinkui’	Total phenolics declined during development; *A. eriantha* retained highest levels, especially gallic acid, conferring superior antioxidant capacity.	Huang et al. [84]
	Sugar–acid balance, vitamin C	Sucrose, glucose, fructose, quinic acid, vitamin C	‘Kuilv’, ‘Hongyang’, ‘Hayward’	Developmental differences shaped nutritional and flavor traits; ‘Kuilv’ high vitamin C, ‘Hongyang’ favorable sugar/acid ratio, ‘Hayward’ higher glucose/fructose.	Li et al. [85]
Postharvest storage	Cold storage metabolomics	Amino acids, fatty acids, organic acids, sugars, vitamin C, phenolics	‘Hayward’, ‘Haegeum’	Cold storage maintained or enhanced antioxidants; cultivar-specific accumulation patterns; metabolic and transcriptomic evidence for slow ripening.	Choi et al. [90]
	VOC dynamics in ready-to-eat fruit	Alcohols, aldehydes, esters, ketones (e.g., ethyl acetate, ethyl butyrate)	‘Xuxiang’	VOCs shifted from aldehydes/alcohols (early) to esters/acids (late); esters linked to sweetness and flavor, aldehydes to firmness loss; optimal consumption at 72–84 h.	Yuan et al. [92]
	Biochemical reprogramming during ripening	Soluble sugars, organic acids, energy metabolites, hormones	‘Jinyan’	Sucrose, glucose, fructose rose; citric acid declined; respiratory intermediates depleted, amino acids accumulated; cytokinin surge in late ripening; enzyme activities correlated with starch breakdown.	Mao et al. [91]
	Temperature effects in kiwiberries	Anthocyanins, flavor-related metabolites	‘Jumbo’, ‘Bingo’, ‘Purpurna Sadowa’	1 °C storage preserved firmness in green cultivars; 6 °C enhanced anthocyanins and flavor compounds in red-fleshed kiwiberries.	Medič et al. [93]
Postharvest treatments	Coating effects on volatiles and quality	78 VOCs (esters, aldehydes, alcohols, methyl salicylate), terpenoids	*A. arguta* (hardy kiwi)	Chitosan and chitosan–silica nanoparticle coatings delayed ripening and preserved quality; CTS-SiNPs shifted aroma toward herbal notes by altering volatile and terpenoid biosynthesis.	Cao et al. [94]

Acronyms: AA = Amino Acids; ADH = Alcohol Dehydrogenase; CAT = Catalase; CTS = Chitosan; CTS-SiNP = Chitosan–Silica Nanoparticle; DEGs = Differentially Expressed Genes; GC-MS = Gas Chromatography–Mass Spectrometry; MDA = Malondialdehyde; POD = Peroxidase; SOD = Superoxide Dismutase; TCA = Tricarboxylic Acid; TF = Transcription Factor; VOC = Volatile Organic Compound; Vit C = Vitamin C.

**Table 4:** Summary of integrated omics studies in kiwifruit outlining major research focuses, key discoveries, and their physiological and postharvest implications.

Theme	Species/Cultivar	Omics Approach	Focus/Trait	Key Findings and Implications	Reference
Reviews & Concept	Fruits including kiwifruit	multi-omics	Postharvest quality, disease defense	Multi-omics can decode ripening, senescence, and stress mechanisms, yet integration remains basic; calls for robust application under real supply-chain conditions and predictive modeling.	Belay and James Caleb [13]
	*Actinidia* spp.	genomics, transcriptomics, proteomics, metabolomics	Breeding & omics integration	Summarizes genomic resources, QTLs, MAS/GS, CRISPR, and omics applications for quality and stress tolerance; provides a roadmap for omics-guided breeding while noting regulatory and adoption barriers.	Nazir et al. [1]
	Fruits including kiwifruit	multi-omics	Postharvest physiology	Identifies biomarkers and molecular networks for chilling, ripening, and treatment responses (Ca, 1-MCP, melatonin); situates kiwifruit within broader postharvest omics advances.	Habibi et al. [14]
Cold/Storage Stress	*A. arguta* (‘KL’, ‘RB’)	Transcriptomics + Metabolomics	Freezing tolerance	Tolerant genotype upregulates flavonoids and trehalose while accumulating lipid degradation markers (LPC/LPE); defines pathways and biomarkers for breeding cold-tolerant lines.	Sun et al. [15]
	*A. chinensis* ‘Hongyang’	Transcriptomics + Metabolomics	Cold-chain breaks	Temperature fluctuations induce ethylene and starch genes but suppress flavonoids; *AcHsfA3a* identified as a key regulator; offers biomarkers for cold-chain management.	Yang et al. [32]
	*A. chinensis* ‘Zesy002’	Multi-omics	Maturity biomarkers for storage	Transcript markers (e.g., *MYB10*, *BAM3.2*, *PMEi*, α-terpineol synthase) with SSC predict storability; enables biomarker-driven harvest and storage decisions.	Favre et al. [41]
	Kiwiberries (*A. arguta* cvs.)	Metabolomics + Physiology	Storage temperature effects	1 °C storage preserves firmness in green cultivars, while 6 °C enhances anthocyanins and flavor compounds in red types; supports cultivar-specific storage strategies.	Medič et al. [93]
Postharvest Treatments	*A. deliciosa* ‘Hayward’	Multi-omics	Calcium dip	Calcium delays ripening by modulating cell-wall, ethylene, and stress pathways (AP2/ERF, NAC); functions as both a structural and signaling modulator.	Polychroniadou et al. [55]
	*A. chinensis* ‘Jinyan’	Transcriptomics + Metabolomics	Chitosan coating	Chitosan slowed softening via suppression of *PG*, *BAM*, and ethylene genes, preserving phenolics and flavonoids; demonstrates CTS as a dual physical and molecular shelf life regulator.	Yang et al. [28]
	*A. arguta*	Transcriptomics + Metabolomics	CTS–SiNP coating	Reduced decay and shifted volatiles toward herbal notes at the cost of sweet/floral aromas; highlights the shelf life vs. aroma trade-off.	Cao et al. [94]
	*A. chinensis* ‘Hongyang’	Transcriptomics + Metabolomics	Ethylene vs. 1-MCP	Ethylene increased sugars and phenolics; 1-MCP preserved acids and slowed ripening; both enhanced polyphenols; supports tuning of ripening and nutritional traits.	Li et al. [26]
Development, Ripening & Pigmentation	*A. chinensis* ‘Hongyang’	Transcriptomics + Metabolomics	Development & ripening (sugars, flavonoids, Vit. C)	Identified 693 metabolites and 47k genes; *AcMYB123-2* and *AcERF192* regulate proanthocyanidins and ascorbate turnover; offers high-resolution gene–metabolite maps for breeding.	Shu et al. [16]
	*A. chinensis* ‘Hongyang’	Transcriptomics + Metabolomics + MALDI-MSI	Color & quality formation	Over 1000 metabolites localized by tissue; anthocyanins in red flesh linked with MYB/bHLH/WD40; demonstrates spatial omics for tissue-specific regulation.	Mao et al. [35]
	*A. chinensis* ‘Hongyang’, ‘Jintao’; *A. arguta* ‘Mini Amethyst’, ‘Kuilv’	Transcriptomics + Metabolomics	Flavonoid/anthocyanin diversity	Pigmented cultivars accumulate more anthocyanins regulated by MYB/bHLH; informs selection for nutritionally enriched types.	Yu et al. [19]
	*A. chinensis* (tetraploid hybrids)	Transcriptomics + Metabolomics	Hybrid nutrition	Tetraploid hybrids show elevated anthocyanins, carotenoids, and vitamin C via upregulation of *CHS*, *DFR*, *ANS*; supports polyploid breeding for nutrition.	Zhang et al. [34]
	*A. chinensis* ‘Hongyang’	Transcriptomics + Metabolomics	Flavor regulation	Identified 34 flavor metabolites associated with *ERF182* and *NAC4*; reveals targets for improving sugars and aroma.	Wang et al. [89]
	*A. chinensis* ‘Jinyan’ (tissues)	Transcriptomics + Metabolomics	Flavonoid biosynthesis	301 flavonoids and >2300 TFs (*bHLH74*, *MYB1R1*, *WRKY33*) regulate tissue-specific profiles; roots and leaves identified as potential nutraceutical sources.	Mao et al. [21]
	*A. chinensis* ‘Jinshi 1’	Transcriptomics + Metabolomics	Color transitions	Early pink coloration from anthocyanins and late yellow from carotenoids via *MYB/bHLH* regulation; defines color breeding mechanisms.	Xiong et al. [88]
	*A. chinensis* ‘Jinshi 1’	Transcriptomics + Metabolomics	Flavor & nutrients	Coordinated control of sugar, acid, and AsA metabolism; *AaSPS* key for ripening sugars; suggests targets for sweetness and AsA enhancement.	Xiong et al. [87]
Nutritional & Stress Traits	*A. eriantha* (‘Ganlv 1’, ‘Ganlv 2’)	Multi-omics + Resequencing	Early maturity	Faster starch–sugar conversion and lower hormone levels; SNPs in candidate genes; provides markers for early-maturity breeding.	Liao et al. [97]
	*A. eriantha* (MM-11/13/16)	Transcriptomics + Metabolomics	Sugar/acid/phenolics/AsA	Cultivar-specific gene–metabolite links (e.g., *GMP*, *MDHAR*, *PAL*, *FLS*); aids breeding for enhanced nutrition.	Jia et al. [20]
	*A. chinensis* vs. *A. eriantha*	Transcriptomics + Metabolomics	Aromatic amino acids	*A. eriantha* enriched in Phe/Trp, while *A. chinensis* higher in serotonin/tryptamine; TFs implicated; clarifies interspecies flavor–nutrition variation.	Hu et al. [2]
	*A. arguta* (‘Qssg’, ‘Lc’)	Transcriptomics + Metabolomics + Bioassay	Flavonoids & hypouricemia	Chrysin, rutin, and daidzein reduce uricemia in mice; biosynthetic genes mapped; supports functional food applications.	Wang et al. [36]
Proteomics & Ripening	*A. deliciosa* ‘Hayward’	Proteomics + Metabolomics	Postharvest ripening	Over 4000 proteins identified; BAMs regulate starch breakdown, ASP3 drives amino acid conversion; defines proteo-metabolomic ripening map.	Tian et al. [24]
	*A. deliciosa* (‘Hayward’, ‘Garmrok’)	Proteomics	Ethylene-regulated ripening	Ethylene alters defense and photosynthetic proteins with cultivar-specific responses; proteomic validation of ethylene-driven ripening.	Shin et al. [95]
Breeding Resources	*Actinidia* spp. (33 accessions; 55 assemblies)	Pan-genomics + Integrated transcriptomes	KPGD platform	Graph pan-genome containing 2.37 M genes (85,940 families) with integrated SNPs/indels/SVs and 1071 RNA-seq datasets; establishes a landmark resource for comparative genomics and precision breeding.	Li et al. [42]

Acronyms: 1-MCP = 1-Methylcyclopropene; AcHsfA3a = Heat Shock Transcription Factor A3a; ANS = Anthocyanidin Synthase; AP2/ERF = APETALA2/Ethylene Response Factor; AsA = Ascorbic Acid; BAM = β-Amylase; bHLH = Basic Helix–Loop–Helix; Ca = Calcium; CCD = Carotenoid Cleavage Dioxygenase; CHS = Chalcone Synthase; CTS = Chitosan; CTS–SiNP = Chitosan–Silica Nanoparticle; DFR = Dihydroflavonol 4-Reductase; ERF = Ethylene Response Factor; FLS = Flavonol Synthase; GMP = Glutamate Monophosphate Dehydrogenase; GS = Genomic Selection; KPGD = Kiwifruit Pan-Genome Database; LPC = Lysophosphatidylcholine; LPE = Lysophosphatidylethanolamine; MALDI-MSI = Matrix-Assisted Laser Desorption Ionization–Mass Spectrometry Imaging; MAS = Marker-Assisted Selection; MDHAR = Monodehydroascorbate Reductase; MYB = Myeloblastosis Transcription Factor; NAC = NAM–ATAF–CUC Transcription Factor; PAs = Proanthocyanidins; PAL = Phenylalanine Ammonia-Lyase; PG = Polygalacturonase; PMEi = Pectin Methylesterase Inhibitor; QTL = Quantitative Trait Locus; SNP = Single-Nucleotide Polymorphism; SPS = Sucrose Phosphate Synthase; SV = Structural Variant; TF = Transcription Factor; Trp = Tryptophan; Vit C = Vitamin C; WD40 = WD-Repeat Protein Family; WRKY = WRKY Domain Transcription Factor.

**Table 5:** Summary of recent advances in nondestructive and data-driven technologies for kiwifruit quality evaluation and prediction.

Technology	Cultivar(s)	Target Parameters	Key Findings and Practical Notes	Reference
NIR-HSI (1000–2500 nm) + PLS	‘Fenghuang-1’	Textural properties	Achieved r > 0.9 for hardness, chewiness, and resilience; shear-related traits less reliable due to testing variability	Li et al. [100]
VNIR-HSI + chemometrics	‘Guichang’	SSC, firmness, maturity	R^2^p = 0.896 (SSC), 0.871 (firmness) with 98% maturity accuracy; color prediction is less robust, needs cross-season validation	Shang et al. [101]
FSI vs. HSI + ML	‘Hongyang’	SSC	FSI outperformed HSI (R^2^p = 0.889, RPD = 2.88); small dataset (n = 90) limits model generalization	Xu et al. [125]
VNIR-HSI + SVMR	‘Hayward’	SSC, firmness, ripeness	R^2^p = 0.94 (SSC), 0.878 (firmness); ripeness classification > 91%; validated only on cold-stored fruit	Lee et al. [126]
HSI (400–1000 nm) + PLS	‘Hayward’	SSC, firmness, ripeness class	R^2^ = 0.94 (SSC), 0.92 (firmness); >90% classification accuracy; lab-based only	Benelli et al. [109]
HSI (390–1030 nm) + visualization	‘Guichang’	SSC, firmness, color	RPD = 2.3–3.1 with ripening visualization maps; single-cultivar validation	Meng et al. [111]
Rotational NIR-HSI	Golden kiwifruit	SSC, pH gradients	Mapped SSC variation (R^2^cv = 0.74) and pH gradients; slow and costly system	Ma et al. [110]
Fluorescence-HSI + ML	‘Hongyang’	DM, SSC, firmness, maturity	SSC R^2^ = 0.80; maturity ≈ 92%; single-cultivar test with fluorescence variability	Zou et al. [49]
Bulk optical properties	‘Hongyang’	SSC, firmness	Rp^2^ = 0.97; strong absorption-SSC and scattering-firmness correlations; requires destructive calibration	Tian et al. [103]
Microwave waveguide	‘Hayward’	Firmness	Low-cost, contactless measurement; prototype stage, limited validation	Berardinelli et al. [104]
Impact vibration + ML	‘Hayward’	Firmness, SSC, TA, sensory	≈92% maturity classification; weak SSC/TA prediction	Zhang et al. [105]
Acoustic elasticity + kinetic model	‘Hongyang’	Firmness, shelf life	R^2^ > 0.99 with accurate shelf life prediction; limited dataset	Zhang et al. [106]
Acoustic stiffness + compression	‘SunGold’	Firmness	R^2^ > 0.89 within a season; reduced cross-season consistency	Sneddon et al. [108]
Vibration + RF regression	‘Hayward’	Firmness	R^2^ = 0.96 (RMSE = 0.0125); single-season small dataset	Nouri and Mehdizadeh [107]
Portable LED–photodiode	‘Xuxiang’, ‘Huayou’	Sweetness, firmness	85–91% accuracy within short time (3 s); accuracy lower in ‘Xuxiang’, requires recalibration	Yang et al. [43]
Smartphone RGB + volatiles	‘Xuxiang’	Freshness, SSC, firmness	RGB-GC-MS integration identified 5-day freshness window; tested at room temperature, single cultivar	Li et al. [44]
E-nose + HSI fusion	‘Hayward’	SSC, firmness, TA, ripeness	94% accuracy; fusion improved robustness; validated only in lab	Bakhshipour [50]
Flexible ethylene sensor + RF	‘Hongyang’	Ripeness	97.5% accuracy with wireless sensing; short-term stability test only	Guo et al. [119]
HSI + CNN	‘Hayward’	Bruise detection	98–100% accuracy (unripe); performance declines after ripening	Ebrahimi et al. [112]
Structured HSI + ANN	‘Zespri’	Early bruising	100% accuracy (0–4 days post-impact); tested under controlled conditions	Liang et al. [113]
HSI + LDA/PLSR	‘Hayward’	Gray mold, SSC, firmness	~96% accuracy for *Botrytis cinerea* detection; lab-only validation	Haghbin et al. [114]
VNIR interactance spectroscopy	‘Zesy002’	Chilling injury	Clear separation of sound vs. CI fruit; broader validation required	Wang et al. [6]
Dual-laser VNIR system	‘Zesy002’	Chilling injury severity	85–94% classification accuracy; less sensitive to mild CI	Wang et al. [116]
Laser backscattering imaging	‘SunGold’, ‘Hayward’	Chilling injury	92% accuracy in ‘SunGold’; trichomes reduce accuracy in ‘Hayward’	Yang et al. [117]
X-ray CT + ML	‘Jinyan’	CI, bruising, lignification	AUC > 0.9 for internal defect classification; expensive, low throughput	Wang et al. [118]
VNIR + ML	‘Hayward’	Storability	Reduced soft fruit by 30% after storage; moderate accuracy for mid-class fruit	Li et al. [120]
FT-NIR vs. Kiwifirm	‘Yunhai No. 1’	Firmness	R^2^p = 0.918; outperformed Kiwifirm™; dataset limited to lab samples	Ding et al. [121]
Kinetic + microbial models	‘Xuxiang’	Shelf life	Predicted ≤ 100 days at 0 °C; lower accuracy for microbial growth	Niu et al. [122]
YOLOv5 object detection	‘Xu Xiang’, ‘Cui Xiang’	Surface defects	97.7% real-time detection; restricted to automated lines	Wang et al. [46]
ResNet34 + CBAM	‘Xu Xiang’, ‘Cui Xiang’	External defects	99.6% accuracy; needs large-scale industrial datasets	Wang et al. [45]
CNN + vibration	Multiple	Hardness classes	98.9–100% accuracy; possible overfitting from limited training	Yang et al. [47]
Physics-data fusion model	‘Hayward’	Flesh firmness	Improved interpretability over PLSR/SVR; assumes static storage	Xiao and Li [123]
Multi-omics framework	‘Hayward’	DM, ripening, phenolics	Identified DEGs linked to starch and phenolic metabolism; no sensor integration	Valasiadis et al. [124]
Data fusion (VNIR-HSI + FT-NIR)	‘Jintao’	SSC, firmness, DM, hue	R^2^ = 0.84–0.91 with 16% error reduction vs. single models; moderate dataset, needs cross-season validation	Cevoli et al. [48]

Acronyms: ANN = Artificial Neural Network; AS = Acoustic Stiffness; AUC = Area Under Curve; CARS = Competitive Adaptive Reweighted Sampling; CBAM = Convolutional Block Attention Module; CNN = Convolutional Neural Network; CT = Computed Tomography; DM = Dry Matter; EI = Elasticity Index; ELM = Extreme Learning Machine; FF = Flesh Firmness; FSI = Fluorescence Spectral Imaging; FT-NIR = Fourier Transform Near-Infrared; GA = Genetic Algorithm; GC-MS = Gas Chromatography–Mass Spectrometry; HSI = Hyperspectral Imaging; LBI = Laser Backscattering Imaging; LDA = Linear Discriminant Analysis; ML = Machine Learning; MLR = Multiple Linear Regression; MSC = Multiplicative Scatter Correction; NIR = Near-Infrared; PCA = Principal Component Analysis; PLS/PLSR = Partial Least Squares/Partial Least Squares Regression; PLS-DA = Partial Least Squares Discriminant Analysis; RF = Random Forest; RPD = Residual Predictive Deviation; SG = Savitzky–Golay Filter; SKNN = Simplified k-Nearest Neighbor; SNV = Standard Normal Variate; SSC = Soluble Solids Content; SVM/SVR/SVMR/SVMC = Support Vector Machine/Support Vector Regression/SVM Regression/SVM Classifier; TA = Titratable Acidity; TLNN = Three-Layer Neural Network; TRS = Time-Resolved Spectroscopy; VIP = Variable Importance in Projection; VNIR = Visible–near-infrared; YOLO = You Only Look Once.

## Data Availability

No new data were created or analyzed in this study.
